# Exploring the Biological and Chemical Complexity of the Ligases

**DOI:** 10.1016/j.jmb.2014.03.008

**Published:** 2014-05-15

**Authors:** Gemma L. Holliday, Syed Asad Rahman, Nicholas Furnham, Janet M. Thornton

**Affiliations:** European Molecular Biology Laboratory, European Bioinformatics Institute, Wellcome Trust Genome Campus, Hinxton, Cambridge CB10 1SD, UK

**Keywords:** EC, Enzyme Commission, NTP, nucleoside triphosphate, NDP, nucleoside diphosphate, NMP, nucleoside monophosphate, MDA, multiple domain architecture, AAM, atom–atom mapping, CoA, coenzyme A, CSA, Catalytic Site Atlas, ligase, overall reaction similarity, enzyme evolution, enzyme function

## Abstract

Using a novel method to map and cluster chemical reactions, we have re-examined the chemistry of the ligases [Enzyme Commission (EC) Class 6] and their associated protein families in detail. The type of bond formed by the ligase can be automatically extracted from the equation of the reaction, replicating the EC subclass division. However, this subclass division hides considerable complexities, especially for the C–N forming ligases, which fall into at least three distinct types. The lower levels of the EC classification for ligases are somewhat arbitrary in their definition and add little to understanding their chemistry or evolution. By comparing the multi-domain architecture of the enzymes and using sequence similarity networks, we examined the links between overall reaction and evolution of the ligases. These show that, whilst many enzymes that perform the same overall chemistry group together, both convergent (similar function, different ancestral lineage) and divergent (different function, common ancestor) evolution of function are observed. However, a common theme is that a single conserved domain (often the nucleoside triphosphate binding domain) is combined with ancillary domains that provide the variation in substrate binding and function.

## Highlights

• The ligases are a small set of enzymes with functions that are critical for life. Their functions include (but are not limited to) the attachment of amino acid residues onto the corresponding tRNA, DNA repair and coenzyme A decorations.
• The subclass level of the EC number, namely, the type of bond formed between the two substrates, is the primary discriminator between the overall chemical transformations. Further detailed examination can produce interesting insights but does not offer more in the means of more detailed automatic classification.
• Many examples exist of ligases that have evolved both divergently and convergently, with examples of ligases that have evolved to work on different substrates using the same chemistry and occasional examples where there is an apparent change of chemistry in the reaction mechanism itself. We show a single example (the acyl-coenzyme A superfamily) in which divergent evolution has occurred to perform different overall bond formations utilising the same basic mechanistic approach.
• Our systematic and automatic approach to comparing and analysing enzyme reactions and the evolution of the associated enzyme sequences provides a robust route to better understand how these ligase enzymes have evolved all the complex chemistries needed for life in diverse environment.

## Graphical abstract

**Figure:**
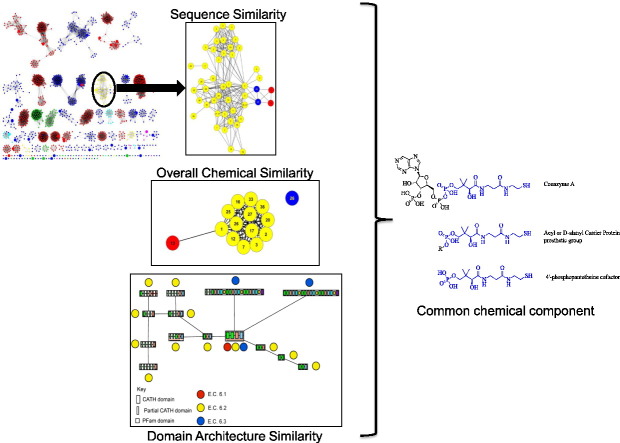

## Introduction

**Fig. 1:**
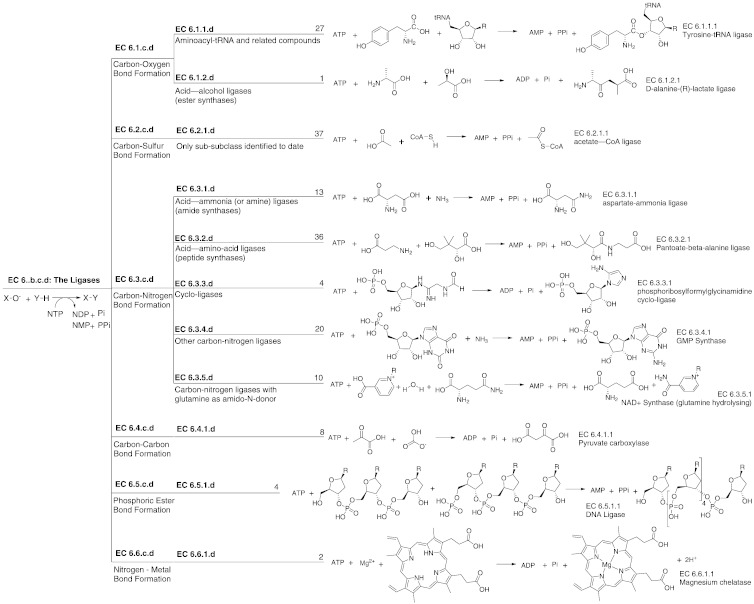
The hierarchical classification of the ligases as defined by the EC. The generic reaction for the ligase enzymes is shown at the far right, each split of the EC classification is represented as a tree, with a brief description of what the split represents. The numbers shown at the end of the branch represent the number of current EC numbers in each sub-subclass and the chemical reaction shown is a single example of an overall transformation in that sub-subclass.

Enzymes have been divided into six basic classes as defined by the Enzyme Commission (EC) [1]. The six classes are the oxidoreductases, transferases, hydrolases, lyases, isomerases and ligases. There are currently (February 2014) 5294 overall chemical transformations (as identified by the EC number) defined. The ligase class is the focus of this paper and is responsible for joining two molecules together with the concomitant hydrolysis of a nucleoside triphosphate (NTP) to either a nucleoside diphosphate (NDP) or a nucleoside monophosphate (NMP). In the majority of cases (158 EC numbers), the NTP is ATP; however, guanosine triphosphate is seen in five cases, cytosine triphosphate is seen in one and one of the DNA ligases uses NAD^+^. Some examples of the different chemistries performed by this class of enzyme are shown in Fig. 1, which provides a broad overview of the EC classification for the ligases.

This class performs many biologically essential reactions; at least 81 ligases are involved in central metabolism (see Fig. S1). Examples of essential functions include the aminoacyl-tRNA synthetases that add the correct amino acid onto the appropriate tRNA molecule required for protein synthesis; the enzymes that repair damaged DNA and RNA are often ligases, as are many of the enzymes that decorate coenzyme A (CoA) with various different acyl groups; glutamine synthetase (EC 6.3.1.2) fixes ammonia in higher plants [2]; carbamyl phosphate synthetase is involved in the removal of excess ammonia in humans and is a key step in pyrimidine and arginine biosynthesis in prokaryotes and eukaryotes [3]. There are over 60 human diseases associated with polymorphisms in ligases (see Table S1), including various cancers (e.g., breast, cervical and liver), epilepsy, hyperammonemia (caused by an enzyme deficiency in the Krebs Cycle), neonatal pulmonary hypertension and mental disorders (many ligase-based disorders lead to reduced metal development).

However, it is also the smallest class of enzymes with only 167 different reactions currently defined by the EC [1]. In comparison, the largest class of enzymes are the transferases with 1567 active EC numbers.

The EC number is a four-number code in the form a.b.c.d, where a is the class of enzyme, b and c respectively represent the subclasses and sub-subclasses and the final number broadly describes the substrate specificity. Generally the first three numbers describe the general overall chemistry being performed. However, underneath this simple classifier, there are nuances that offer further insight into the ligase enzymes, including the hydrolysis products of the NTP and the more detailed descriptions of the reactive centres involved. However, chemistry is only one part of the picture; the biology (in the form of protein sequence and structure) is also a key component in understanding this class of enzyme. However, linking chemistry with biology is a significant challenge.

In this paper, we seek to provide an overview of the ligase class of enzyme by analysing their reactions and considering the structure and evolution of the proteins that perform the chemistry. We utilise a novel software tool, EC-BLAST† [4], which allows the automatic comparison and characterisation of chemical reactions according to the bond changes involved, the substrate and product substructure similarity and the similarity of the reaction centres.

## The Overall Chemistry of the Ligase Class

The EC classification splits the ligases into six subclasses (Fig. 1), defined according to the type of bond being formed. The sub-subclass (third level of the EC number) defines the type of substrate involved; however, only two subclasses are currently further divided: these ligases that form a C–O (EC 6.1.c.d) or a C–N bond (EC 6.3.c.d).

### Reaction data

Of the 167 ligases with a defined EC number, only 133 have an available, fully balanced reaction in the last freely available release of the KEGG database [5] (Release 58.1, June 2011). This highlights one of the challenges that we have faced in performing this analysis: the paucity of data. This is seen not only in the absence of complete and/or balanced reactions (e.g., many of the DNA ligases that repair nicks in the DNA backbone lack reaction files in KEGG due to the difficulty in representing the substrates accurately) but also in the lack of biological data. Only 108 of the ligases (based on EC number) analysed have one or more sequences deposited in the manually curated section of UniProtKB [6], and only 75 also have at least one associated crystal structure in the wwPDB [7]. At the time this paper went into revision (February 2014), there are 30,582 proteins in the reviewed section of UniProtKB that are classified as ligases, with 583 unique proteins having an associated structure, covering 135 and 96 EC numbers, respectively. There are 34 enzymes for which we have information on the active site in the Catalytic Site Atlas (CSA) [8] and 21 enzymes where we have mechanistic information in MACiE [9]. Where possible, errors and inconsistencies have been corrected and we have used a manually curated dataset in the analysis presented here.

### Clustering overall reactions

#### By covalent bond changes

**Fig. 2:**
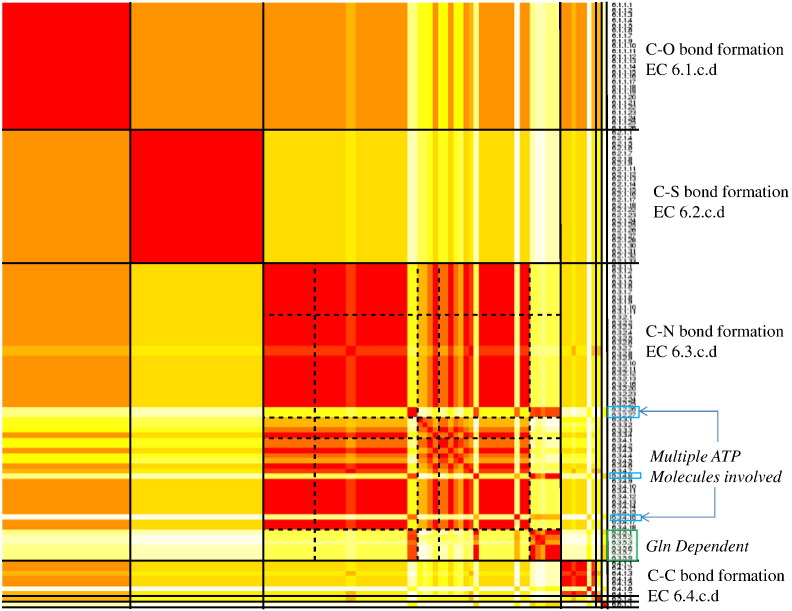
Heat map generated using the R statistical package showing the similarity of the overall transformations in the ligase class, ordered by EC number, based on the bond changes occurring. Similarities are shown from red to white with red representing a similarity score of 1 (i.e., identical) and white representing a similarity score of 0 (i.e., completely different). The broken lines indicate the sub-subclasses in the EC 6.3 C–N forming subclass of the ligases. (For a full list of the EC numbers represented in this heat map, please see Table S2.)

Once the 133 reactions were processed (see Materials and Methods) and the atom–atom mapping (AAM) was completed, we used EC-BLAST to create three different fingerprints to characterise the bond changes, the reaction centres involved and the substructures of the substrates and products for each reaction. We performed an all-against-all comparison of the overall reactions according to the changes in the covalent bonds occurring during the course of the reactions and calculated a similarity matrix of all the reactions to one another (Fig. 2). Then, we used the EC classification as a “gold standard” to which we compared our results. Figure 2 shows the similarity matrix as a heat map in which the similarities between bond changes in reactions are ordered by EC number. This shows that the bond changes are captured reliably, re-creating the subclass level of the EC classification with the various subclasses being clearly distinguished from one another.

In some subclasses, for example, the C–O (EC 6.1.c.d) and C–S (EC 6.2.c.d) bond forming enzymes, all the enzymes make or break the same bonds and are uniformly identical by this criterion.

In contrast, the C–N bond forming ligases (EC 6.3.c.d) show significant complexities and this subclass is split into three groups: the “simple” group that forms a C–N bond without any attendant complex bond changes (such as in stereochemistry or involving double bonds); the “complex” C–N bond formations that commonly have attendant changes in double bonds, often the cleavage of a C = O double bond or formation of a C = N double bond; and finally, the glutamine-dependent ligases (6.3.5.d) that use the hydrolysis of glutamine to glutamate to produce the required ammonia molecule.

Several of the “complex” C–N bond forming reactions [6.3.2.26, 6.3.2.27 (which has recently been deleted and replaced with two separate EC numbers 6.3.2.38 and 6.3.2.39) and 6.3.4.16; highlighted in blue in Fig. 2] all involve multiple ATP molecules and the joining of more than two molecules, performing several rounds of reaction in the same active site. They look very similar to one another with respect to the overall reaction bond changes and appear significantly different to the rest of the subclass. EC 6.3.4.8 (also highlighted in blue, in Fig. 2) looks very similar to the enzymes that include multiple ATP molecules, as the second substrate is 5-phospho-alpha-d-ribose-1-diphosphate. In this reaction, the ribose portion is ligated onto the imidazole-4-acetate substrate and the second product is the diphosphate moiety.

In the lower section of the heat map are those enzymes that are the only representatives of their subclass, as is the case with the phosphoric ester (6.5.c.d) and nitrogen–metal (EC 6.6.c.d) bond forming ligases. In the first case, this is because the enzymes are responsible for fixing broken DNA and RNA, reactions that are hard to represent in small-molecule format. Although there are four well-characterised enzymes in this subclass, only one is represented in KEGG. In the latter case, only two EC numbers are assigned to this sub-subclass and only one was available from KEGG at the time of this analysis.

#### By reaction centre and substructure similarity

The reaction similarity heat map presented in Fig. 2 is based solely upon the similarity of the bond changes between the overall reactions, but the reaction centres around those bond changes and the substrates involved may be very different. In addition to bond changes, EC-BLAST permits the comparison of both reaction centres and molecular substructures describing a given reaction. The reaction centres are captured by the covalent chemistry (atoms and bonds) surrounding each of the bond changes and the substructures of both substrates and products are captured as a composite molecular fingerprint (see Materials and Methods). Thus, for discrimination between ligases with the same bond change characteristics, we can further cluster such reactions based upon their reaction centres and the substructure similarity of the substrates and products.

**Fig. 3:**
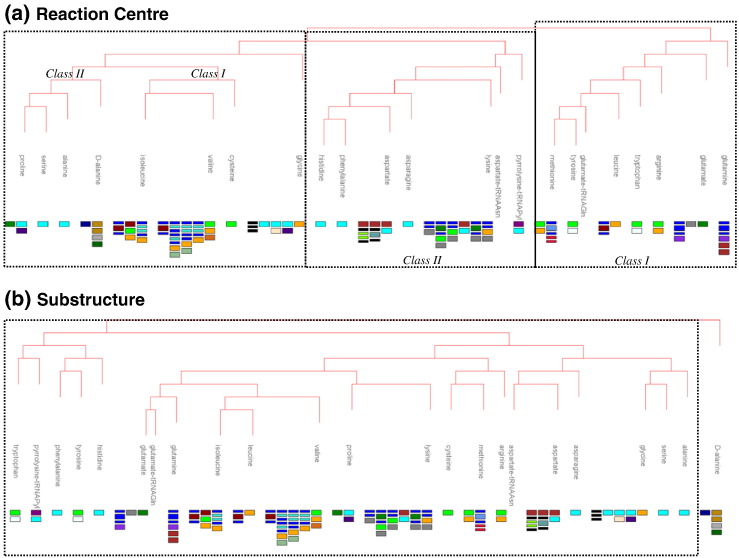
Similarity of (a) the reaction centre and (b) the substructure for the ligase reactions in the EC subclass of 6.1 (carbon–oxygen bond forming). The statistically significant subclusters are shown in the broken boxes. The leaves of the tree are annotated with the name of the amino acid substrate involved and the known multi-domain architectures as represented by CATH domain composition, shown using rectangles and coloured such that the same domain is always shown in the same colour; a slender rectangle denotes a partial domain.

As an example, the reaction centre and substructure similarity trees for the C–O bond forming subclass (EC 6.1.c.d) are shown in Fig. 3a and b, respectively. This C–O bond forming subclass is dominated by the numerous aminoacyl-tRNA ligases, which join an amino acid to its appropriate tRNA. There is, however, one other enzyme in this subclass [d-alanine—poly(phosphoribitol) ligase; EC 6.1.1.13] that does not have tRNA as a substrate (this enzyme will be discussed in more detail below).

It is well established that there are two types of tRNA synthases. Class I tRNA ligases acylate the 2′-OH of the terminal ribose and the active site contains a Rossmann dinucleotide binding domain (CATH domain 3.40.50.620, represented by the orange rectangles in Fig. 3) with the ATP typically binding in an extended conformation. Class II tRNA ligases acylate the 3′-OH of the terminal ribose and the active site contains an anti-parallel beta-fold (CATH domain 3.30.930.10, represented by the cyan rectangles in Fig. 3) with the ATP molecule typically binding in a bent conformation [10,11]. There is no observable difference between the overall bond changes for the two types of tRNA ligase, as can be seen in Fig. 2; however, when the reaction centre is used to cluster the EC numbers, there is a marked difference between the two types (see Fig. 3a). Here, the enzymes, labelled according to the amino acid involved in the reaction, are clustered into three statistically significant groupings, two of which clearly correspond to the Class I and Class II division, whilst the third contains both Class I and Class II enzymes. This third group contains those enzymes that utilise amino acid residues with no C^γ^ or a branched C^β^_,_ and there are clearly three subsets: the singleton glycyl-tRNA ligase (officially a Class II tRNA ligase; however, glycine is unique amongst the amino acid residues in that it has no C^β^), then into the Class I tRNA ligases (which includes the non-tRNA utilising amino acid enzyme) and Class II enzymes.

The lysyl-tRNA synthetase (LysRS; EC 6.1.1.6) is a case where a single EC number is represented by two distinct classes of aminoacyl-tRNA ligase. These two enzymes are not related through divergent evolution but are related through functional convergence [12]. Historically, it was assumed that Achaea lacked a LysRS gene but work by Ibba et al.[13] showed that ^14^C-labelled lysine was incorporated into proteins of *Methanococcus maripaludis*, proving that there was indeed a protein that performed the LysRS function. However, this protein showed no similarity to any other of the known LysRS proteins (which are of the Class I type) and was in fact more similar to the Class II type proteins.

In Fig. 3b, the enzymes are clustered by the substructure similarity of their substrates generating a very different tree structure, for example, the small amino acids cluster on the right-hand side of the tree whilst the large aromatic molecules cluster on the left. Looking at the reactions in this way clearly identifies the singlet non-tRNA containing reaction as an outlier but does not differentiate between the two distinct types of aminoacyl-tRNA ligase.

It is clear from this that there is no one way of looking at the data; orthogonal data types reveal different features and the combination of these makes for a more complete picture. Thus, for the EC 6.1 subclass, both types of clustering are valuable and reveal different properties of the reactions.

However, not all subclasses behave so cleanly, even at the overall bond level, for example, the C–N forming ligases (EC subclass 6.3.c.d; see Fig. 2). For this subclass level, it is especially clear that, just because two EC numbers are numerically adjacent, their reactions are not necessarily similar. This is due to the fact that the fourth digit of the EC number, usually referred to as a serial number, discriminates between the many different substrates and products involved. However, it is assigned sequentially in time and therefore carries no information about the chemical similarity of molecules involved.

In the EC 6.3.c.d C–N bond forming subclass, clustering the data by reaction centre broadly splits the enzyme into the NDP forming, NMP forming, those utilising NH_3_ and those utilising more than one ATP (data not shown). However, there are many statistically significant splits, which result in relatively small groupings in which there are usually only one or two enzymes. Further, clustering by the structures of the molecules involved generates few clearly defined groupings, saving those enzymes that utilise the same substrate or products. For example, enzymes that utilise biotin as a substrate are clearly clustered together (see Fig. S2a). However, in one enzyme, the reaction is ligating the biotin onto another protein, and in the other, it is adding carbon dioxide to the biotin molecule. Thus, the two enzymes are acting on very different parts of the biotin molecule with different reaction centres, as can be seen in the lack of grouping of these enzymes by the reaction centre clustering (see Fig. S2b).

Another example of C–N bond forming enzymes that perform very similar reactions is the carbamyl phosphate synthetases [14] (EC 6.3.5.5 and 6.3.4.16). Both EC numbers represent the same basic chemical transformation, the addition of an ammonia molecule to a bicarbonate molecule, the only difference being the source of the ammonia. In the case of EC 6.3.5.5, the ammonia comes from glutamine (the enzyme has an associated glutamine hydrolase domain, either as part of the complex or as a fusion protein); in the case of EC 6.3.4.16, the ammonia is taken in by the protein directly. Furthermore, there are three classes of carbamyl phosphate synthetase known: Class I is found in mitochondria and involved in the urea cycle; Class II is found in the cytosol and involved in pyrimidine metabolism; Class III is currently only identified in fish. In this case, it is likely that the core mechanism is almost identical between the enzymes, but the addition of the extra domain changes not only the ultimate source of the ammonia but also the overall reaction.

## Mechanism in the Ligases

**Fig. 4:**
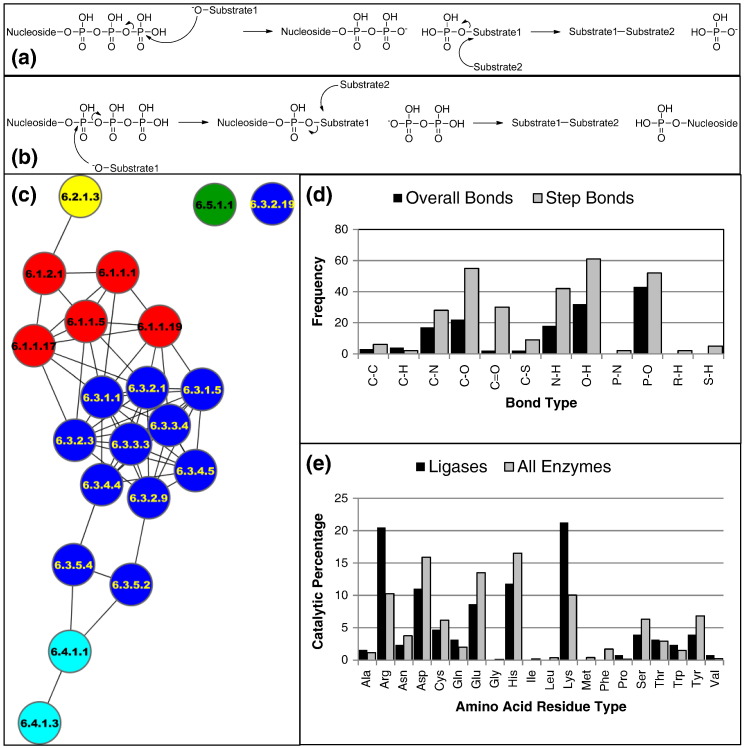
The mechanistic details of the ligase reactions. (a) The mechanistic pattern where the gamma phosphate of the NTP is attacked that leads to the formation of NDP and Pi. (b) The mechanistic pattern where the alpha phosphate of the NTP is attacked, which leads to the formation of NMP and PPi. (c) The mechanism similarity of the 21 ligases in MACiE 3.0 determined by the composite bond change, measured for each step in the reaction and summed. An edge is drawn at a similarity value of 0.5 or greater. (d) The sum of the bond changes involved in the steps (black) and overall (grey) reactions. (e) The percentage of catalytic residues for each amino acid residue type in the ligase class (black) and all enzymes in MACiE 3.0 and the CSA V. 2.0 (grey).

All ligases perform their function using a broadly similar mechanism (see Fig. 4a and b) with three or fewer steps, which can be described as the initial activation of the substrate, followed by the addition of the substrate onto the gamma (Fig. 4a) or alpha phosphate (Fig. 4b) of the NTP. The second substrate then displaces the nucleoside portion, forming the bond by which the subclass is named. Further differences are found in the nature of the NTP utilised. Whilst ATP is the most common, both guanosine triphosphate and cytosine triphosphate have been observed as substrates. There is also a ligase [EC 6.5.1.2, DNA ligase (NAD^+^)] in which the hydrolysed molecule is not an NTP, but NAD^+^, resulting in products of adenosine monophosphate (AMP) and beta-nicotinamide d-ribonucleotide.

In the vast majority of cases, at least one of the substrates in the ligase class is an organic acid; the carboxylate group usually undergoes a nucleophilic substitution to one of the phosphate groups of the NTP. The molecule to which this acid is concatenated onto is one of the following: an alcohol (O–H group, 6.1.c.d and 6.4.c.d), a thiol (S–H group, 6.2.c.d) or an amine (N–H group, 6.3.c.d and 6.6.c.d). This second substrate adds onto the carbonyl carbon of the newly formed phosphoric ester, cleaving a C–O bond. The cleavage of this C–O bond is clearly reflected in the bond change profile of the ligase class (Fig. 4d) as is the fact that, in all cases, there is at least one P–O and O–H bond broken and formed.

The 19 enzymes for which mechanisms are available in MACiE 3.0 have been clustered by the composite bond changes, measured for each step of their reactions and summed (see Fig. 4c). As in Fig. 2, the ligases within one subclass cluster together, with some outliers, especially in the C–N class. Here, the glutamine-dependent ligases (EC 6.3.5.d) have different mechanisms compared to the other enzymes in this class due to their requirement to generate ammonia from the glutamine before the C–N bond can be formed.

However, in MACiE, there are also two enzymes that have a distinctly different mechanism (unconnected nodes in Fig. 4c) to the rest of the class (these enzymes are not included in EC-BLAST due to their absence in the version of KEGG used). In these two examples (EC 6.3.2.19, ubiquitin transfer cascade and EC 6.5.1.1, DNA ligase), there is a nucleophilic amino acid residue in the active site (Cys and Lys, respectively). In the case of the ubiquitin transfer cascade, the Cys residue is responsible for the transfer of a ubiquitin group from one part of the enzyme to another. In the case of the DNA ligase, the Lys residue activates the AMP molecule for attachment to the first DNA molecule, and the bond formed as part of the ligation is between a phosphorus and oxygen, which distinguishes it from the rest of the class as there is no carbon atom involved.

In general, the residues in ligase active sites are mostly responsible for the activation and stabilisation of the substrates and reactive intermediates. Thus, it is difficult to state with any certainty that there are specific residues acting with respect to specific chemistries, agreeing with our previous observations [15]. Strikingly, the positively charged Arg and Lys residues are the most frequent catalytic residues in the ligase class, and in both cases, they are over-represented compared to the distribution of residues in the complete set of enzymes held in MACiE and the CSA (Fig. 4e). Gln is also over-represented although this residue is much rarer in the dataset than either Arg or Lys. These distributions reflect the need to stabilise the negatively charged phosphate groups, which are ubiquitous in all the ligases.

## Domain Structure of the Ligases

**Fig. 5:**
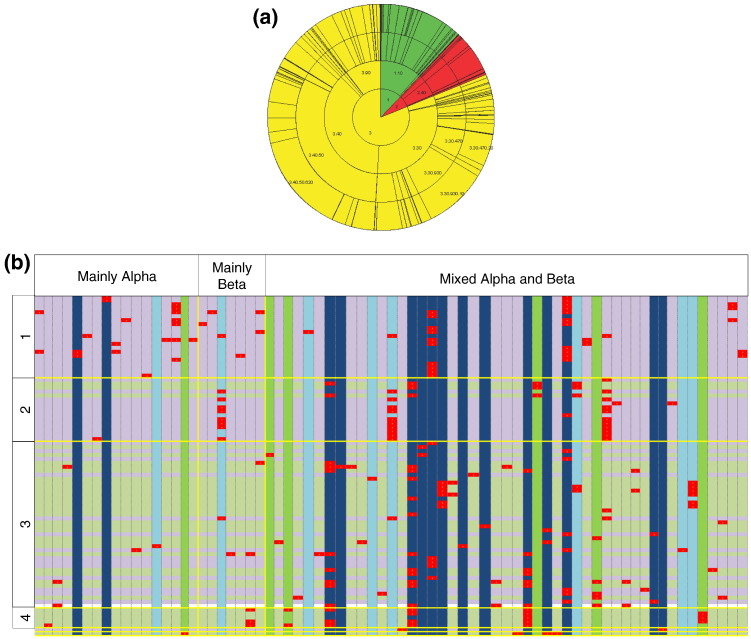
(a) CATH Wheel showing the diversity of CATH domains associated with the ligase class. Mainly alpha (green); mainly beta (red); alpha plus beta (yellow). The numbers shown in the segments are CATH numbers, representing class, architecture, topology and homologous family. (b) The co-occurrence of CATH domains with EC numbers for the ligases. Here, the EC numbers are represented in the rows and the CATH domains are in the columns (see Supplementary Material for the table version of this plot). Lilac rows represent those EC numbers that form NMP, pale green represents those that form NDP and, if the NTP hydrolysis product is unknown, the row is white. Dark-blue columns are those CATH domains that are both NTP binding and catalytic, pale-blue columns are NTP binding only and green columns are those domains that are catalytic only. Red cells indicate that this combination of CATH domain and EC number has been observed in ligases.

Ligase reactions are performed by many different unrelated domains, including the mainly alpha, mainly beta and mixed alpha and beta structures, with the latter predominating (Fig. 5a), which is a common pattern for all enzymes.

Figure 5b shows the co-occurrence of different structural domains, described using the CATH classification system (based on the structural class, architecture, topology and homologues superfamily) (columns) with EC numbers (rows). All observed domains with a given enzyme function (EC number) are shown with a red cell. The full table is included in Supplementary Material 2 as an Excel spreadsheet. The summation row and column in this full table show that some domains are only associated with one enzyme function, some are confined to a single enzyme subclass (i.e., form only one type of bond) and others occur with multiple functions, forming different types of bonds. Likewise, some functions are performed by only one domain, whilst others are performed by multiple unrelated domains.

It is interesting to note that, for the nucleotide (NTP) binding domains, there appears to be an exclusive correspondence between the specific domain and the type of NTP hydrolysis product (e.g., ADP or ATP), as illustrated by the lilac and light-green background row colouring. It is also these binding domains that tend to be present in multiple EC subclasses (i.e., have multiple enzyme functions). Many of the enzymes incorporate multiple different domains, as illustrated for the C–O bond forming ligases in Fig. 3a.

The wide representation of domains from all the major classes in the CATH classification combined with the variety of multi-domain architectures found within the ligases indicates that evolution of the critical chemistry has occurred through both modulation of molecular features in the active site and combinations of domains driving the functional diversity [16]. This occurs through both divergent (where enzyme sequences and structures diverge over time from a common ancestor to perform different functions with preservation of the mechanism, e.g., in the case of the acyl-CoA ligases discussed in detail below) and convergent (where completely unrelated enzymes converge to perform the same function often with very different mechanisms, e.g., in the case of the lysyl-tRNA synthetases discussed earlier) evolution. The presence of many so-called ancient domains in the ligases, which are found across all kingdoms of life, suggests that the diversification of overall chemistry and function occurred very early in evolutionary history.

In Fig. 3 (which only shows EC subclass 6.1.c.d), the various different minimal multiple domain architectures (MDAs) are shown for each of the enzymes performing the different overall reactions. From this, it is clear that the same reaction can be performed by many different proteins often with quite different MDAs; for example, the proline-tRNA ligase and methionine-tRNA ligase can be considered to be examples of convergent evolution. Others have a range of minimal MDAs but share common domains (shown by the same coloured rectangle). Likewise, the same domain can be common to many different enzymes. There are several possible explanations for this observation, including enzyme promiscuity through lack of substrate specificity, minimal enzymatic involvement in the catalytic mechanism (i.e., the enzyme's role is limited to binding and stabilising the substrates and intermediates, but is not acting as a covalent catalyst, thus removing the need to fully conserve any one catalytic motif) and domain evolution. This will be discussed in more detail below.

## Sequence Similarity between the Ligases

**Fig. 6:**
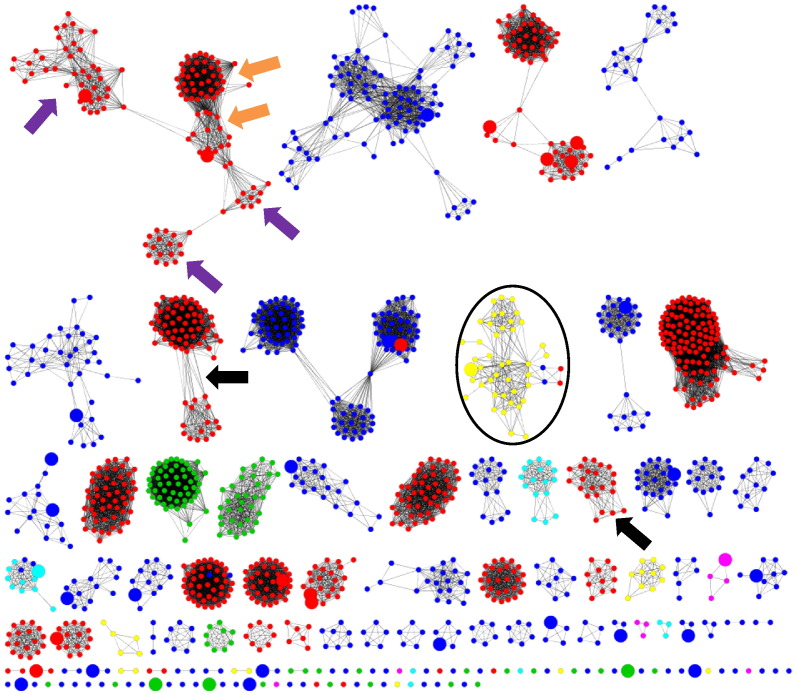
Sequence similarity representative network for the ligase enzymes at an *E*-value of 1 × 10^− 30^, coloured by EC subclass: red nodes represent C–O bond forming (6.1.c.d), yellow nodes represent C–S bond forming (6.2.c.d), blue nodes represent the C–N bond forming (6.3.c.d), cyan nodes represent the C–C bond forming (6.4.c.d), green nodes represent the P–O bond forming (6.5.c.d) and magenta nodes represent the nitrogen–metal bond forming (6.6.c.d) ligases. Image generated using the organic layout algorithm in Cytoscape [17]. Large nodes represent the presence of a crystal structure. The network was created using Cytoscape and shown in the organic layout. The cluster of interest is shown in a black oval. The black arrows represent the two evolutionarily distinct chains associated with the EC 6.1.1.20. The orange arrows represent the type I aminoacyl-tRNA synthases and the purple arrows represent the type II aminoacyl-tRNA synthases.

Since structural data are sparse, an alternative and informative way of examining the general trends of evolution in the ligases is to use sequence similarity. Such an approach may fail to reveal very distance relationships seen by structural comparison, but the advantage of having many more sequences is beneficial. All the ligase sequences with good annotation [i.e., annotated in the manually curated section of UniProtKB (Swiss-Prot)] are clustered using standard approaches to create a representative sequence similarity network (see Materials and Methods for details) and coloured by their subclass membership (Fig. 6).

In the majority of cases, clustering at the *E*-value cutoff of 1 × 10^− 30^ reveals little evolutionary transfer from one subclass function (i.e., bond type formed) to another. The C–O and C–N bond forming ligases, unsurprisingly, dominate the sequence similarity network as these are some of the most common bonds involved in the small-molecule chemistries performed by enzymes. Whilst most of the clusters appear to maintain a single EC number throughout, there is a non-trivial number that contains multiple EC numbers. For example, the EC 6.1.c.d cluster at the top left of the figure contains both Class I (indicated by an orange arrow in Fig. 6) and Class II (indicated by a purple arrow in Fig. 6) aminoacyl-tRNA synthases. However, other EC numbers, for example, EC 6.1.1.20 (phenylalanine—tRNA ligase, clusters annotated with a black arrow in Fig. 6), are seen in two distinct clusters that represent the two distinct protein chains needed for the enzyme to be active. However, the diversity seen in the aminoacyl-tRNA ligases (Fig. 3), including in the variety of multi-domain architectures, where the primary chemical difference is in the reaction centre and the amino acid is involved, suggests a long history *via* many different evolutionary routes for this critical class of protein.

## Evolving Chemical Function: Changing between C–S, C–O and C–N bond formation

**Fig. 7:**
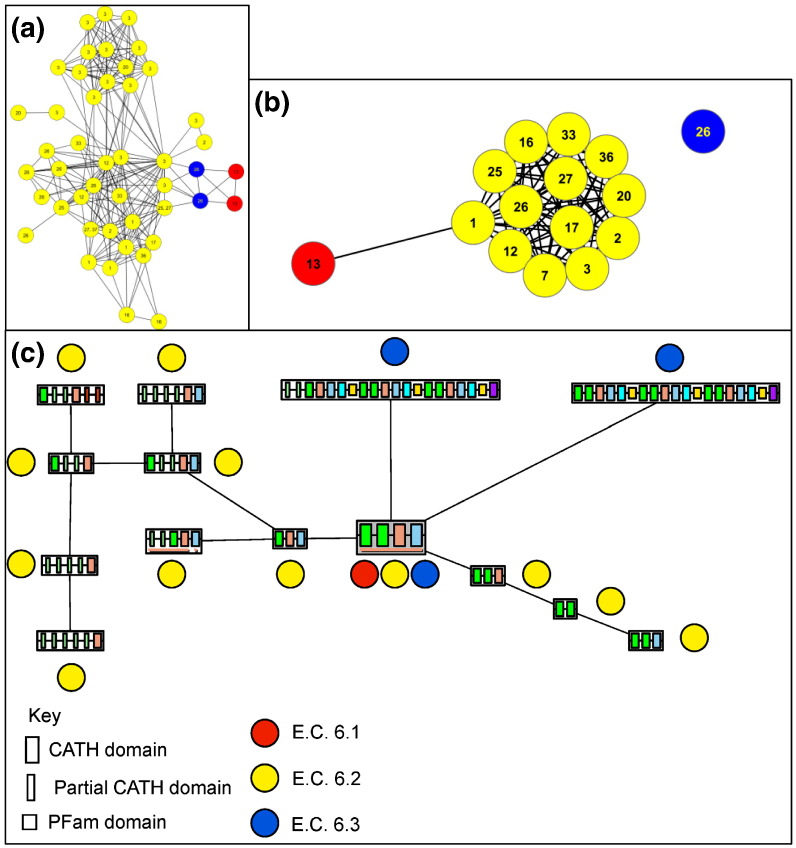
Acyl-CoA ligase group. (a) Representative sequence similarity network of the cluster of interest at an *E*-value of 1 × 10^− 30^. Image generated using the organic layout algorithm in Cytoscape [17]. This cluster includes 15 distinct EC numbers. (b) Overall reaction similarity network where similarity is calculated as the reactive centre and bond change similarity at a Tanimoto score of 0.5. Image generated using the organic layout algorithm in Cytoscape [17]. (c) Multi-domain architecture network of the cluster of interest. In all cases, the numbers written in the nodes represent the serial numbers of the ligases contained. Yellow nodes represent the C–S ligases (EC 6.2.c.d), red nodes represent the C–O ligases (EC 6.1.c.d) and blue nodes represent the C–N ligases (EC 6.3.c.d).

However, there are some clusters that include one or more enzymes forming a different overall bond from the majority of the cluster. The most notable example is the acyl-CoA ligases. This chemically diverse cluster, highlighted with a black oval in Fig. 6 and shown enlarged in Fig. 7a, includes the enzymes listed, along with their overall reactions, in Table S3. The majority of the sequences perform C–S bond formation (EC 6.2.1.d, shown in yellow in Fig. 7) between CoA and a range of substrates as defined by the variety of serial numbers in the annotated EC numbers. In addition, there are sequences that catalyse two different bond formations: C–O [EC 6.1.1.13, d-alanine—poly(phosphoribitol) ligase, shown in red in Fig. 7] and C–N [EC 6.3.2.26, *N*-(5-amino-5-carboxypentanoyl)-l-cysteinyl-d-valine synthase, shown in blue in Fig. 7]. This poses the question: what evolutionary changes have permitted these related sequences to perform seemingly quite different chemistries?

From the plot of the associated multi-domain architectures and their respective functions (see Fig. 7c), it can be seen that there is one MDA that performs all three bond forming functions. This four domain architecture is clearly functionally diverse, forming C–O, C–S and C–N bonds. However, the C–S bond forming enzymes (EC 6.2.c.d) adopt several different MDAs with different domain compositions. This suggests that a smaller protein may well be a competent 6.2.c.d enzyme and that the C–S bond formation is relatively simple with a simple domain architecture. There are several higher-order MDAs that also perform the *N*-(5-amino-5-carboxypentanoyl)-l-cysteinyl-d-valine synthase (EC 6.3.2.26) function. Each of these contains three repeats of the four-domain core that performs all three bond type formations, along with other decorations. This suggests that this change of function has occurred by modulation of the active-site residues, rather than by domain accretion.

Structural data are available for a number of the sequences with EC 6.2.1.d functions that share the common four-domain architecture and a structure for a sequence with d-alanine—poly(phosphoribitol) ligase (EC 6.1.1.13) function [18] (see Fig. S3). Using these structures, it is possible to build a model of the sequence with *N*-(5-amino-5-carboxypentanoyl)-l-cysteinyl-d-valine synthase (EC 6.3.2.26) function (data not shown). Though the pairwise percentage sequence identities are less than 20%, the sequence alignment and structural modelling were straightforward, apart from two large insertions in EC 6.3.2.26 away from the active-site region that could not be modelled. There are a number of motifs [19] conserved in all three sequences and structures involved in catalysis and substrate binding (see Supplementary Material).

Although these two enzymes (6.1.1.13 and 6.3.2.26) appear to have very different substrates and final products, a review of the literature [18,19] shows that there are some striking similarities between these and the more numerous CoA ligases (EC 6.2.c.d) (see Supplementary Material for more details). In both cases, there is an intermediate step in which a C–S bond is formed. Further, in all the enzymes in this set, the sulfur at which the ligation occurs is found as part of the 4′-phosphopantetheine moiety (Fig. S7). Thus, the conservation of structure and sequence motifs conserves the chemistry in the form of a single step (or a partial mechanism) between the different functions. Hence, the change in EC number hides the common use of these activated substrates in different contexts, which gives rise to a change in the overall reaction (see Supplementary Material for more detail). This is similar to the observation made by Bartlett *et al.* [20] in their study of 27 pairs of related enzymes with different functions. In the majority of pairs, they found that the chemistry was in fact conserved for part of the reaction mechanism but that this was often decorated with additional steps at the beginning, middle or end of the reactions, as seen in the ligase case where there are decorations both before and after the defining C–S bond formation.

## Conclusions and Future Outlook

The ligases are a small set of enzymes with functions that are critical for life. The ability to join molecules by the formation of different bonds is the primary discriminator between different ligase reactions and is probably the best way to classify the ligases into different groups. This defines the subclass in the EC nomenclature and this can now be characterised automatically with EC-BLAST. Further discrimination, especially for C–N bond forming ligases, highlights enzymes that form multiple and complex C–N bonds. Whilst further subclustering can produce interesting insights, either by reaction centre or by substructure, the clusters garnered from such an effort are usually too small and the results are too complex to be of much use in creating a new, single, automated classification system for ligases.

The EC number was only ever designed to classify the overall chemical transformation that an enzyme performs. Despite the fact that it is manual and thus prone to human error, it does this remarkably well. However, enzymes have many more attributes than just their overall chemistry; thus, it is essential that when we investigate these biological machines, we take into account not only the chemistry they perform but also the sequence and structural information that is available. However, the evolution of sequence, structure and function occurs at very different rates. It only takes a minute change (sometimes only one amino acid residue) to alter the chemical function of an enzyme, whilst in other cases, the sequence can diverge significantly and the chemistry remains the same.

A significant challenge that remains is the identification of an enzyme's function from its sequence [21] or from mechanism [22]. The most successful approach to function identification utilises the idea of an enzyme superfamily [23]. Here, the evolutionarily relatedness of a group of enzymes usually results in the conservation of some active-site features and some aspect of the chemical reaction. By linking a sequence to a superfamily, it becomes possible to infer something about its function. However, many superfamilies are multi-functional across many distinct EC classes (e.g., the radical SAM, enolase and amidohydrolase superfamilies, to name a few) making prediction particularly difficult for members of these families. Thus, a holistic approach, such as provided in this paper, is needed. However, we are (as ever) somewhat limited to the current state of knowledge, which is far behind the amount of data available to the community.

As with all enzymes, we find many examples where ligases have evolved to work on different substrates using the same chemistry and occasional examples where there is an apparent change of chemistry in the reaction mechanism itself. Also some chemical reactions are performed by two completely unrelated enzymes with different mechanisms. A full study of evolution within each family is needed to reveal all the different types of molecular changes that give rise to the different enzyme functions. In addition, more functional experimental data are required to provide a strong experimental basis for any conclusions. However, our systematic and automatic approach to comparing and analysing enzyme reactions and the evolution of the associated enzyme sequences provides a robust route to better understand how these ligase enzymes have evolved all the complex chemistries needed for life in diverse environments.

## Materials and Methods

### Dataset and reaction processing

The ligase reactions were gathered from the last freely available version of KEGG (Release 58.1, June 2011) [5]. Each reaction file was cross-checked for being balanced at the molecular level (the number and type of atoms in the reactant side is equal to the number and type of atoms in the products). The AAM and bond change annotation of these reaction files was then performed using EC-BLAST^2^. The AAM algorithm composes of matrix manipulation and graph theoretical models complemented by branch and bound techniques to choose a set of chemically feasible mapping solutions. The best mapping solutions will generate one-to-one correspondence between the atoms of the reactant and product molecules and minimise the bond/energy changes and in effect, the chaos in the system. Once mapped, the reactions can be processed to determine their similarity to one another.

A further complication is the quality of the reaction data, specifically with respect to the way in which the stereochemistry is depicted; whilst many of the molecules have the correct stereochemistry, the translation of a three-dimensional reality into a two-dimensional representation is fraught with difficulties, and most atom-mapping software is not designed to handle stereochemistry. We have overcome many of the complications, but there are still cases where the stereochemical changes are erroneous. However, the methods we have utilised in this study have allowed us to identify errors in the EC classification (such as in reaction R01164, the EC number is ADP forming whilst the KEGG reaction is AMP forming) and representational challenges, for example, where one annotator has represented a complex molecule one way, and another has done so differently, for example, the aminoacyl-tRNA ligases where one set of tRNA molecules are depicted with a terminal adenine base and the other set is with a generic R group. Whilst both representations are correct, they can result in very different chemical similarities which are potentially spurious. Thus, we have endeavoured to represent such cases in a consistent manner where possible and used a manually curated dataset for the analysis presented here.

#### Fingerprints

The mapping and the bond changes based on the outcome of the previous strategy is then selected for the reaction fingerprint generation. We compute three types for chemical fingerprints, namely:

(i) Bond change fingerprint: This consists of bonds broken formed/broken, order change and atom stereo changes in a reaction.
(ii) Reaction centre: Surrounding atoms and bonds around the bond changes are computed as circular fingerprints or signatures.
(iii) Structural fingerprints: This is a weighted hashed fingerprint‡ of the substrate and products moieties involved in the reactions.

These fingerprints are transformed into fixed length vectors using the hashing code algorithm and the similarity between them is computed using Jaccard's coefficient. The similarity score ranges from “0” to “1” where the former represents dissimilarity and the latter represents similarity. An all-by-all similarity measure between all the reactions involved in the ligase class can be transformed into a similarity matrix of size M × M where M is the number of reactions in the ligase class. The similarity matrix was then clustered using either *k*-means or hierarchical clustering method such as PVClust§. In PVClust, *p*-values are calculated *via* multi-scale bootstrap resampling. The R statistical∥ computing package was used for clustering these matrices.

#### Further processing of the reaction data

The *k*-means clustering of the bond changes results in a heat map and initial identification of statistically significant groupings of reactions. These groups were further re-clustered using the PVClust hierarchical clustering on the basis of the reaction centre similarity or structural similarity. The clusters from the later method with *p* ≥ 0.95 were highly likely to be classified together based on their reaction pattern similarity scores.

### Sequence similarity networks

Taking all the sequences in the manually annotated portion of UniProtKB (Swiss-Prot) [6] that are annotated as being a ligase (21,953 sequences from the enzyme.dat file as of August 2012), we used the Pythoscape framework [24] to generate the initial representative network. Firstly, the all-against-all BLAST scores are calculated for the initial protein set. Then, the protein sequences are clustered into groups of similar proteins (40% sequence similarity was used in this study) using Cd-hit [25]. This reduces the complexity of the network to be analysed and resulted in 1519 representative nodes where each representative node can contain one or more individual sequences. The representative similarity network is then produced by calculating the similarity between the nodes as the mean − log10(*E*-value) of all the pairwise alignments between all sequences abstracted by each representative node.

### Attributing functional domains within a multi-domain architecture

Enzymes may consist of many different structural domains, one or more of which may be absolutely required for catalytic activity. However, there may also be domains associated with an enzyme that are not part of the function of interest. For example, a protein may be a fusion of two domains, each performing different overall chemical transformations. In such a case, the only domain we are interested in is the one that is directly associated with our function of interest. However, such information is not trivial to access as the protein sequence is often annotated as a whole and thus as performing both functions.

Thus, we use the concept of the minimal multi-domain architecture as the minimal domain, or combination of domains, required to perform the enzyme's function. To identify this, we use a combinatorial approach. Each multi-domain architecture associated with a specific function, defined by the EC number, as collected from the CATH [26] and CATH-Gene3D [27] resource (v3.5). Any unassigned sequence regions large enough to constitute a domain are checked against Pfam [28], and if a non-overlapping Pfam domain is found, then it is included in the multi-domain architecture. From this pool of different multi-domain architectures, the smallest combination of domains is searched for in the largest domain combination. If this smaller multi-domain architecture is found within the larger domain combination, then the larger multi-domain architecture is removed from the pool. Once the first iteration has finished, the next smallest domain combination is used as the search object. This process is repeated until all domain combinations have been searched for within the other larger domain combinations. The result of this searching is a unique list of the minimal domain/multi-domain architectures that can perform the same function. The domains that were present but subsequently removed are called “confusion domains” and assumed not to be critical to the function being investigated, although in practise, they may well modulate the function.

## Footnotes

† http://www.ebi.ac.uk/thornton-srv/software/rbl/

‡ http://chembioinfo.com/2011/10/30/revisiting-molecular-hashed-fingerprints/

§ http://www.is.titech.ac.jp/~shimo/prog/pvclust/

∥ http://www.r-project.org/
