# Supplementary material for: Exploring the Biological and Chemical Complexity of the Ligases
Source: J Mol Biol. 2014 May 15;426(10):2098–111. doi: 10.1016/j.jmb.2014.03.008 (PMC4018984; doi:10.1016/j.jmb.2014.03.008)
Supplement: Supplementary file 1 — Supplementary material 1 [file mmc1.docx]

**Supplementary Material**


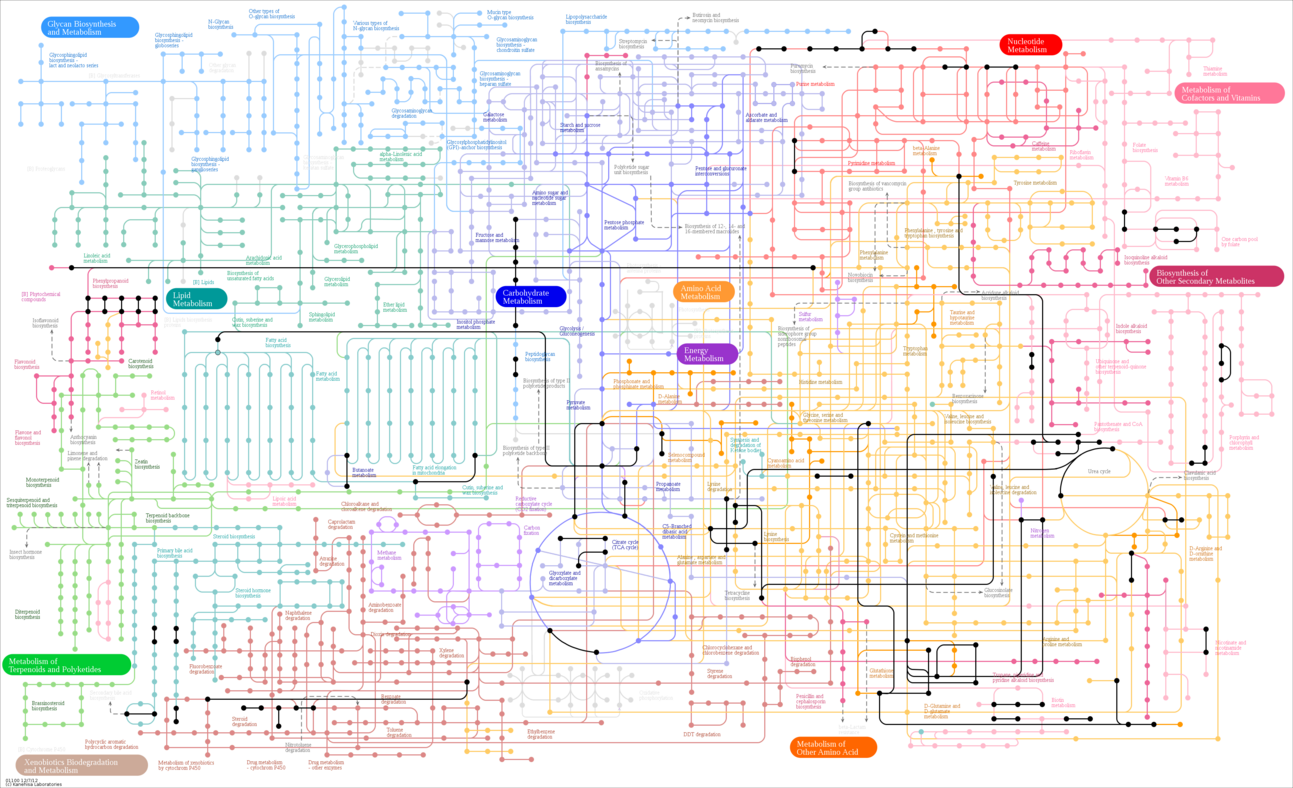


*Figure S1: Central Metabolism Pathway image from KEGG (map:01100) with the ligase reactions coloured in black.*

| ***Entry*** | ***EC number*** | ***Disease*** | ***Protein names*** |
| --- | --- | --- | --- |
| O15164 | 6.3.2.- | Thyroid papillary carcinoma (TPC) [MIM:188550]: A common tumor of the thyroid that typically arises as an irregular, solid or cystic mass from otherwise normal thyroid tissue. Papillary carcinomas are malignant neoplasm characterized by the formation of numerous, irregular, finger-like projections of fibrous stroma that is covered with a surface layer of neoplastic epithelial cells. | Transcription intermediary factor 1-alpha (TIF1-alpha) (EC 6.3.2.-) (E3 ubiquitin-protein ligase TRIM24) (RING finger protein 82) (Tripartite motif-containing protein 24) |
| O15344 | 6.3.2.- | Opitz GBBB syndrome 1 (OGS1) [MIM:300000]: A congenital midline malformation syndrome characterized by hypertelorism, genital-urinary defects such as hypospadias in males and splayed labia in females, cleft lip/palate, laryngotracheoesophageal abnormalities, imperforate anus, developmental delay and congenital heart defects. | E3 ubiquitin-protein ligase Midline-1 (EC 6.3.2.-) (Midin) (Putative transcription factor XPRF) (RING finger protein 59) (RING finger protein Midline-1) (Tripartite motif-containing protein 18) |
| O60260 | 6.3.2.- | Parkinson disease (PARK) [MIM:168600]: A complex neurodegenerative disorder characterized by bradykinesia, resting tremor, muscular rigidity and postural instability. Additional features are characteristic postural abnormalities, dysautonomia, dystonic cramps, and dementia. The pathology of Parkinson disease involves the loss of dopaminergic neurons in the substantia nigra and the presence of Lewy bodies (intraneuronal accumulations of aggregated proteins), in surviving neurons in various areas of the brain. The disease is progressive and usually manifests after the age of 50 years, although early-onset cases (before 50 years) are known. The majority of the cases are sporadic suggesting a multifactorial etiology based on environmental and genetic factors. However, some patients present with a positive family history for the disease. Familial forms of the disease usually begin at earlier ages and are associated with atypical clinical features. Parkinson disease 2 (PARK2) [MIM:600116]: A neurodegenerative disorder characterized by bradykinesia, rigidity, postural instability, tremor, and onset usually before 40. It differs from classic Parkinson disease by early DOPA-induced dyskinesia, diurnal fluctuation of the symptoms, sleep benefit, dystonia and hyper-reflexia. Dementia is absent. Pathologically, patients show loss of dopaminergic neurons in the substantia nigra, similar to that seen in Parkinson disease; however, Lewy bodies (intraneuronal accumulations of aggregated proteins) are absent. | E3 ubiquitin-protein ligase parkin (EC 6.3.2.-) (Parkinson juvenile disease protein 2) (Parkinson disease protein 2) |
| O60488 | 6.2.1.3 | Mental retardation, X-linked 63 (MRX63) [MIM:300387]: A disorder characterized by significantly below average general intellectual functioning associated with impairments in adaptive behavior and manifested during the developmental period. Intellectual deficiency is the only primary symptom of non-syndromic X-linked mental retardation, while syndromic mental retardation presents with associated physical, neurological and/or psychiatric manifestations. Alport syndrome with mental retardation, midface hypoplasia and elliptocytosis (ATS-MR) [MIM:300194]: A X-linked contiguous gene deletion syndrome characterized by glomerulonephritis, sensorineural hearing loss, mental retardation, midface hypoplasia and elliptocytosis. | Long-chain-fatty-acid--CoA ligase 4 (EC 6.2.1.3) (Long-chain acyl-CoA synthetase 4) (LACS 4) |
| O94972 | 6.3.2.- | Mulibrey nanism (MUL) [MIM:253250]: Autosomal recessive disorder that involves several tissues of mesodermal origin, implying a defect in a highly pleiotropic gene. Characteristic features include severe growth failure of prenatal onset and constrictive pericardium with consequent hepatomegaly. In addition, muscle hypotonia, J-shaped sella turcica, yellowish dots in the ocular fundi, typical dysmorphic features and hypoplasia of various endocrine glands causing hormonal deficiency are common. | E3 ubiquitin-protein ligase TRIM37 (EC 6.3.2.-) (Mulibrey nanism protein) (Tripartite motif-containing protein 37) |
| O95363 | 6.1.1.20 | Combined oxidative phosphorylation deficiency 14 (COXPD14) [MIM:614946]: A severe multisystemic autosomal recessive disorder characterized by neonatal onset of global developmental delay, refractory seizures, and lactic acidosis. Biochemical studies show deficiencies of multiple mitochondrial respiratory enzymes. | Phenylalanine--tRNA ligase, mitochondrial (EC 6.1.1.20) (Phenylalanyl-tRNA synthetase) (PheRS) |
| P00966 | 6.3.4.5 | Citrullinemia 1 (CTLN1) [MIM:215700]: The classic form of citrullinemia, an autosomal recessive disease characterized primarily by elevated serum and urine citrulline levels. Ammonia intoxication is another manifestation. It is a disorder of the urea cycle, usually manifesting in the first few days of life. Affected infants appear normal at birth, but as ammonia builds up in the body they present symptoms such as lethargy, poor feeding, vomiting, seizures and loss of consciousness. Less commonly, a milder form can develop later in childhood or adulthood. | Argininosuccinate synthase (EC 6.3.4.5) (Citrulline--aspartate ligase) |
| P05165 | 6.4.1.3 | Propionic acidemia type I (PA-1) [MIM:606054]: Life-threatening disease characterized by episodic vomiting, lethargy and ketosis, neutropenia, periodic thrombocytopenia, hypogammaglobulinemia, developmental retardation, and intolerance to protein. | Propionyl-CoA carboxylase alpha chain, mitochondrial (PCCase subunit alpha) (EC 6.4.1.3) (Propanoyl-CoA:carbon dioxide ligase subunit alpha) |
| P05166 | 6.4.1.3 | Propionic acidemia type II (PA-2) [MIM:606054]: Life-threatening disease characterized by episodic vomiting, lethargy and ketosis, neutropenia, periodic thrombocytopenia, hypogammaglobulinemia, developmental retardation, and intolerance to protein. | Propionyl-CoA carboxylase beta chain, mitochondrial (PCCase subunit beta) (EC 6.4.1.3) (Propanoyl-CoA:carbon dioxide ligase subunit beta) |
| P09936 | 3.4.19.12; 6.-.-.- | Parkinson disease 5 (PARK5) [MIM:613643]: A complex neurodegenerative disorder with manifestations ranging from typical Parkinson disease to dementia with Lewy bodies. Clinical features include parkinsonian symptoms (resting tremor, rigidity, postural instability and bradykinesia), dementia, diffuse Lewy body pathology, autonomic dysfunction, hallucinations and paranoia. | Ubiquitin carboxyl-terminal hydrolase isozyme L1 (UCH-L1) (EC 3.4.19.12) (EC 6.-.-.-) (Neuron cytoplasmic protein 9.5) (PGP 9.5) (PGP9.5) (Ubiquitin thioesterase L1) |
| P11161 | 6.3.2.- | Neuropathy, congenital hypomyelinating or amyelinating (CHN) [MIM:605253]: A severe degenerating neuropathy that results from a congenital impairment in myelin formation. It is clinically characterized by early onset of hypotonia, areflexia, distal muscle weakness, and very slow nerve conduction velocities (as low as 3m/s). Some patients manifest nearly complete absence of spontaneous limb movements, respiratory distress at birth, and complete absence of myelin shown by electron microscopy of peripheral nerves. Inheritance can be autosomal dominant or recessive. Charcot-Marie-Tooth disease 1D (CMT1D) [MIM:607678]: A dominant demyelinating form of Charcot-Marie-Tooth disease, a disorder of the peripheral nervous system, characterized by progressive weakness and atrophy, initially of the peroneal muscles and later of the distal muscles of the arms. Charcot-Marie-Tooth disease is classified in two main groups on the basis of electrophysiologic properties and histopathology: primary peripheral demyelinating neuropathies (designated CMT1 when they are dominantly inherited) and primary peripheral axonal neuropathies (CMT2). Demyelinating neuropathies are characterized by severely reduced nerve conduction velocities (less than 38 m/sec), segmental demyelination and remyelination with onion bulb formations on nerve biopsy, slowly progressive distal muscle atrophy and weakness, absent deep tendon reflexes, and hollow feet. Dejerine-Sottas syndrome (DSS) [MIM:145900]: A severe degenerating neuropathy of the demyelinating Charcot-Marie-Tooth disease category, with onset by age 2 years. Characterized by motor and sensory neuropathy with very slow nerve conduction velocities, increased cerebrospinal fluid protein concentrations, hypertrophic nerve changes, delayed age of walking as well as areflexia. There are both autosomal dominant and autosomal recessive forms of Dejerine-Sottas syndrome. | E3 SUMO-protein ligase EGR2 (EC 6.3.2.-) (AT591) (Early growth response protein 2) (EGR-2) (Zinc finger protein Krox-20) |
| P11498 | 6.4.1.1 | Pyruvate carboxylase deficiency (PC deficiency) [MIM:266150]: Leads to lactic acidosis, mental retardation and death. It occurs in three forms: mild or type A, severe neonatal or type B, and a very mild lacticacidemia. | Pyruvate carboxylase, mitochondrial (EC 6.4.1.1) (Pyruvic carboxylase) (PCB) |
| P12081 | 6.1.1.21 | Usher syndrome 3B (USH3B) [MIM:614504]: A syndrome characterized by progressive vision and hearing loss during early childhood. Some patients have the so-called 'Charles Bonnet syndrome,' involving decreased visual acuity and vivid visual hallucinations. USH is a genetically heterogeneous condition characterized by the association of retinitis pigmentosa with sensorineural deafness. Age at onset and differences in auditory and vestibular function distinguish Usher syndrome type 1 (USH1), Usher syndrome type 2 (USH2) and Usher syndrome type 3 (USH3). USH3 is characterized by postlingual, progressive hearing loss, variable vestibular dysfunction, and onset of retinitis pigmentosa symptoms, including nyctalopia, constriction of the visual fields, and loss of central visual acuity, usually by the second decade of life. | Histidine--tRNA ligase, cytoplasmic (EC 6.1.1.21) (Histidyl-tRNA synthetase) (HisRS) |
| P14373 | 6.3.2.- | Thyroid papillary carcinoma (TPC) [MIM:188550]: A common tumor of the thyroid that typically arises as an irregular, solid or cystic mass from otherwise normal thyroid tissue. Papillary carcinomas are malignant neoplasm characterized by the formation of numerous, irregular, finger-like projections of fibrous stroma that is covered with a surface layer of neoplastic epithelial cells. | Zinc finger protein RFP (EC 6.3.2.-) (RING finger protein 76) (Ret finger protein) (Tripartite motif-containing protein 27) |
| P14868 | 6.1.1.12 | Hypomyelination with brainstem and spinal cord involvement and leg spasticity (HBSL) [MIM:615281]: An autosomal recessive leukoencephalopathy characterized by onset in the first year of life of severe spasticity, mainly affecting the lower limbs and resulting in an inability to achieve independent ambulation. Affected individuals show delayed motor development and nystagmus; some may have mild mental retardation. Brain MRI shows hypomyelination and white matter lesions in the cerebrum, brainstem, cerebellum, and spinal cord. | Aspartate--tRNA ligase, cytoplasmic (EC 6.1.1.12) (Aspartyl-tRNA synthetase) (AspRS) (Cell proliferation-inducing gene 40 protein) |
| P15104 | 6.3.1.2; 4.1.1.15 | Congenital systemic glutamine deficiency (CSGD) [MIM:610015]: Rare developmental disorder with severe brain malformation resulting in multi-organ failure and neonatal death. Glutamine is largely absent from affected patients serum, urine and cerebrospinal fluid. | Glutamine synthetase (GS) (EC 6.3.1.2) (Glutamate decarboxylase) (EC 4.1.1.15) (Glutamate--ammonia ligase) |
| P15918 | 3.1.-.-; 6.3.2.- | Combined cellular and humoral immune defects with granulomas (CHIDG) [MIM:233650]: Immunodeficiency disease with granulomas in the skin, mucous membranes, and internal organs. Other characteristics include hypogammaglobulinemia, a diminished number of T and B cells, and sparse thymic tissue on ultrasonography. Severe combined immunodeficiency autosomal recessive T-cell-negative/B-cell-negative/NK-cell-positive (T(-)B(-)NK(+) SCID) [MIM:601457]: A form of severe combined immunodeficiency (SCID), a genetically and clinically heterogeneous group of rare congenital disorders characterized by impairment of both humoral and cell-mediated immunity, leukopenia, and low or absent antibody levels. Patients present in infancy recurrent, persistent infections by opportunistic organisms. The common characteristic of all types of SCID is absence of T-cell-mediated cellular immunity due to a defect in T-cell development. Omenn syndrome (OS) [MIM:603554]: Severe immunodeficiency characterized by the presence of activated, anergic, oligoclonal T-cells, hypereosinophilia, and high IgE levels. Alpha/beta T-cell lymphopenia, with gamma/delta T-cell expansion, severe cytomegalovirus infection and autoimmunity (T-CMVA) [MIM:609889]: An immunological disorder characterized by oligoclonal expansion of TCR gamma/delta T-cells, TCR alpha/beta T-cell lymphopenia, severe, disseminated cytomegalovirus infection and autoimmune cytopenia. | V(D)J recombination-activating protein 1 (RAG-1) (RING finger protein 74) [Includes: Endonuclease RAG1 (EC 3.1.-.-); E3 ubiquitin-protein ligase RAG1 (EC 6.3.2.-)] |
| P22681 | 6.3.2.- | Noonan syndrome-like disorder with or without juvenile myelomonocytic leukemia (NSLL) [MIM:613563]: A syndrome characterized by a phenotype reminiscent of Noonan syndrome. Clinical features are highly variable, including facial dysmorphism, short neck, developmental delay, hyperextensible joints and thorax abnormalities with widely spaced nipples. The facial features consist of triangular face with hypertelorism, large low-set ears, ptosis, and flat nasal bridge. Some patients manifest cardiac defects. Some have an increased risk for certain malignancies, particularly juvenile myelomonocytic leukemia. | E3 ubiquitin-protein ligase CBL (EC 6.3.2.-) (Casitas B-lineage lymphoma proto-oncogene) (Proto-oncogene c-Cbl) (RING finger protein 55) (Signal transduction protein CBL) |
| P31327 | 6.3.4.16 | Carbamoyl phosphate synthetase 1 deficiency (CPS1D) [MIM:237300]: An autosomal recessive disorder of the urea cycle causing hyperammonemia. It can present as a devastating metabolic disease dominated by severe hyperammonemia in neonates or as a more insidious late-onset condition, generally manifesting as life-threatening hyperammonemic crises under catabolic situations. Clinical features include protein intolerance, intermittent ataxia, seizures, lethargy, developmental delay and mental retardation. Pulmonary hypertension, neonatal (PHN) [MIM:615371]: A disease characterized by elevated pulmonary artery pressure. Pulmonary hypertension in the neonate is associated with multiple underlying problems such as respiratory distress syndrome, meconium aspiration syndrome, congenital diaphragmatic hernia, bronchopulmonary dysplasia, sepsis, or congenital heart disease. | Carbamoyl-phosphate synthase [ammonia], mitochondrial (EC 6.3.4.16) (Carbamoyl-phosphate synthetase I) (CPSase I) |
| P38398 | 6.3.2.- | Breast cancer (BC) [MIM:114480]: A common malignancy originating from breast epithelial tissue. Breast neoplasms can be distinguished by their histologic pattern. Invasive ductal carcinoma is by far the most common type. Breast cancer is etiologically and genetically heterogeneous. Important genetic factors have been indicated by familial occurrence and bilateral involvement. Mutations at more than one locus can be involved in different families or even in the same case. Breast-ovarian cancer, familial, 1 (BROVCA1) [MIM:604370]: A condition associated with familial predisposition to cancer of the breast and ovaries. Characteristic features in affected families are an early age of onset of breast cancer (often before age 50), increased chance of bilateral cancers (cancer that develop in both breasts, or both ovaries, independently), frequent occurrence of breast cancer among men, increased incidence of tumors of other specific organs, such as the prostate. Ovarian cancer (OC) [MIM:167000]: The term ovarian cancer defines malignancies originating from ovarian tissue. Although many histologic types of ovarian tumors have been described, epithelial ovarian carcinoma is the most common form. Ovarian cancers are often asymptomatic and the recognized signs and symptoms, even of late-stage disease, are vague. Consequently, most patients are diagnosed with advanced disease. Pancreatic cancer 4 (PNCA4) [MIM:614320]: A malignant neoplasm of the pancreas. Tumors can arise from both the exocrine and endocrine portions of the pancreas, but 95% of them develop from the exocrine portion, including the ductal epithelium, acinar cells, connective tissue, and lymphatic tissue. | Breast cancer type 1 susceptibility protein (EC 6.3.2.-) (RING finger protein 53) |
| P41250 | 6.1.1.14 | Charcot-Marie-Tooth disease 2D (CMT2D) [MIM:601472]: A dominant axonal form of Charcot-Marie-Tooth disease, a disorder of the peripheral nervous system, characterized by progressive weakness and atrophy, initially of the peroneal muscles and later of the distal muscles of the arms. Charcot-Marie-Tooth disease is classified in two main groups on the basis of electrophysiologic properties and histopathology: primary peripheral demyelinating neuropathies (designated CMT1 when they are dominantly inherited) and primary peripheral axonal neuropathies (CMT2). Neuropathies of the CMT2 group are characterized by signs of axonal degeneration in the absence of obvious myelin alterations, normal or slightly reduced nerve conduction velocities, and progressive distal muscle weakness and atrophy. Nerve conduction velocities are normal or slightly reduced. Neuronopathy, distal hereditary motor, 5A (HMN5A) [MIM:600794]: A disorder characterized by distal muscular atrophy mainly affecting the upper extremities, in contrast to other distal motor neuronopathies. These constitute a heterogeneous group of neuromuscular diseases caused by selective degeneration of motor neurons in the anterior horn of the spinal cord, without sensory deficit in the posterior horn. The overall clinical picture consists of a classical distal muscular atrophy syndrome in the legs without clinical sensory loss. The disease starts with weakness and wasting of distal muscles of the anterior tibial and peroneal compartments of the legs. Later on, weakness and atrophy may expand to the proximal muscles of the lower limbs and/or to the distal upper limbs. | Glycine--tRNA ligase (EC 6.1.1.14) (Diadenosine tetraphosphate synthetase) (AP-4-A synthetase) (Glycyl-tRNA synthetase) (GlyRS) |
| P48506 | 6.3.2.2 | Hemolytic anemia due to gamma-glutamylcysteine synthetase deficiency (HAGGSD) [MIM:230450]: A disease characterized by hemolytic anemia, glutathione deficiency, myopathy, late-onset spinocerebellar degeneration, and peripheral neuropathy. | Glutamate--cysteine ligase catalytic subunit (EC 6.3.2.2) (GCS heavy chain) (Gamma-ECS) (Gamma-glutamylcysteine synthetase) |
| P48637 | 6.3.2.3 | Glutathione synthetase deficiency (GSS deficiency) [MIM:266130]: Severe form characterized by an increased rate of hemolysis and defective function of the central nervous system. Glutathione synthetase deficiency of erythrocytes (GLUSYNDE) [MIM:231900]: Mild form causing hemolytic anemia. | Glutathione synthetase (GSH synthetase) (GSH-S) (EC 6.3.2.3) (Glutathione synthase) |
| P49588 | 6.1.1.7 | Charcot-Marie-Tooth disease 2N (CMT2N) [MIM:613287]: An axonal form of Charcot-Marie-Tooth disease, a disorder of the peripheral nervous system, characterized by progressive weakness and atrophy, initially of the peroneal muscles and later of the distal muscles of the arms. Charcot-Marie-Tooth disease is classified in two main groups on the basis of electrophysiologic properties and histopathology: primary peripheral demyelinating neuropathies (designated CMT1 when they are dominantly inherited) and primary peripheral axonal neuropathies (CMT2). Neuropathies of the CMT2 group are characterized by signs of axonal degeneration in the absence of obvious myelin alterations, normal or slightly reduced nerve conduction velocities, and progressive distal muscle weakness and atrophy. Nerve conduction velocities are normal or slightly reduced. | Alanine--tRNA ligase, cytoplasmic (EC 6.1.1.7) (Alanyl-tRNA synthetase) (AlaRS) (Renal carcinoma antigen NY-REN-42) |
| P49590 | 6.1.1.21 | Perrault syndrome 2 (PRLTS2) [MIM:614926]: A sex-influenced disorder characterized by sensorineural deafness in both males and females and ovarian dysgenesis in females. Affected females have primary amenorrhea, streak gonads, and infertility, whereas affected males show normal pubertal development and are fertile. | Probable histidine--tRNA ligase, mitochondrial (EC 6.1.1.21) (Histidine--tRNA ligase-like) (Histidyl-tRNA synthetase) (HisRS) |
| P49792 | 6.3.2.- | Encephalopathy, acute, infection-induced, 3 (IIAE3) [MIM:608033]: A rapidly progressive encephalopathy manifesting in susceptible individuals with seizures and coma. It can occur within days in otherwise healthy children after common viral infections such as influenza and parainfluenza, without evidence of viral infection of the brain or inflammatory cell infiltration. Brain T2-weighted magnetic resonance imaging reveals characteristic symmetric lesions present in the thalami, pons and brainstem. | E3 SUMO-protein ligase RanBP2 (EC 6.3.2.-) (358 kDa nucleoporin) (Nuclear pore complex protein Nup358) (Nucleoporin Nup358) (Ran-binding protein 2) (RanBP2) (p270) |
| P49917 | 6.5.1.1 | LIG4 syndrome (LIG4S) [MIM:606593]: Characterized by immunodeficiency and developmental and growth delay. Patients display unusual facial features, microcephaly, growth and/or developmental delay, pancytopenia, and various skin abnormalities. Severe combined immunodeficiency autosomal recessive T-cell-negative/B-cell-negative/NK-cell-positive with sensitivity to ionizing radiation (RSSCID) [MIM:602450]: A form of severe combined immunodeficiency, a genetically and clinically heterogeneous group of rare congenital disorders characterized by impairment of both humoral and cell-mediated immunity, leukopenia, and low or absent antibody levels. Patients present in infancy with recurrent, persistent infections by opportunistic organisms. The common characteristic of all types of SCID is absence of T-cell-mediated cellular immunity due to a defect in T-cell development. Individuals affected by RS-SCID show defects in the DNA repair machinery necessary for coding joint formation and the completion of V(D)J recombination. A subset of cells from such patients show increased radiosensitivity. | DNA ligase 4 (EC 6.5.1.1) (DNA ligase IV) (Polydeoxyribonucleotide synthase [ATP] 4) |
| P50747 | 6.3.4.-; 6.3.4.9; 6.3.4.10; 6.3.4.11; 6.3.4.15 | Holocarboxylase synthetase deficiency (HLCS deficiency) [MIM:253270]: A neonatal form of multiple carboxylase deficiency, an autosomal recessive disorder of biotin metabolism, characterized by ketoacidosis, hyperammonemia, excretion of abnormal organic acid metabolites, and dermatitis. In holocarboxylase synthetase deficiency, clinical and biochemical symptoms improve dramatically with administration of biotin. | Biotin--protein ligase (EC 6.3.4.-) (Biotin apo-protein ligase) [Includes: Biotin--[methylmalonyl-CoA-carboxytransferase] ligase (EC 6.3.4.9); Biotin--[propionyl-CoA-carboxylase [ATP-hydrolyzing]] ligase (EC 6.3.4.10) (Holocarboxylase synthetase) (HCS); Biotin--[methylcrotonoyl-CoA-carboxylase] ligase (EC 6.3.4.11); Biotin--[acetyl-CoA-carboxylase] ligase (EC 6.3.4.15)] |
| P53597 | 6.2.1.4; 6.2.1.5 | Mitochondrial DNA depletion syndrome 9 (MTDPS9) [MIM:245400]: A severe disorder due to mitochondrial dysfunction. It is characterized by infantile onset of hypotonia, lactic acidosis, severe psychomotor retardation, progressive neurologic deterioration, and excretion of methylmalonic acid. | Succinyl-CoA ligase [ADP/GDP-forming] subunit alpha, mitochondrial (EC 6.2.1.4) (EC 6.2.1.5) (Succinyl-CoA synthetase subunit alpha) (SCS-alpha) |
| P54577 | 6.1.1.1 | Charcot-Marie-Tooth disease, dominant, intermediate type, C (CMTDIC) [MIM:608323]: A form of Charcot-Marie-Tooth disease, a disorder of the peripheral nervous system, characterized by progressive weakness and atrophy, initially of the peroneal muscles and later of the distal muscles of the arms. The dominant intermediate type C is characterized by clinical and pathologic features intermediate between demyelinating and axonal peripheral neuropathies, and motor median nerve conduction velocities ranging from 25 to 45 m/sec. | Tyrosine--tRNA ligase, cytoplasmic (EC 6.1.1.1) (Tyrosyl-tRNA synthetase) (TyrRS) [Cleaved into: Tyrosine--tRNA ligase, cytoplasmic, N-terminally processed] |
| P98170 | 6.3.2.- | Lymphoproliferative syndrome, X-linked, 2 (XLP2) [MIM:300635]: A rare immunodeficiency characterized by extreme susceptibility to infection with Epstein-Barr virus (EBV). Symptoms include severe or fatal mononucleosis, acquired hypogammaglobulinemia, pancytopenia and malignant lymphoma. | E3 ubiquitin-protein ligase XIAP (EC 6.3.2.-) (Baculoviral IAP repeat-containing protein 4) (IAP-like protein) (ILP) (hILP) (Inhibitor of apoptosis protein 3) (IAP-3) (hIAP-3) (hIAP3) (X-linked inhibitor of apoptosis protein) (X-linked IAP) |
| Q00987 | 6.3.2.- |  | E3 ubiquitin-protein ligase Mdm2 (EC 6.3.2.-) (Double minute 2 protein) (Hdm2) (Oncoprotein Mdm2) (p53-binding protein Mdm2) |
| Q05086 | 6.3.2.- | Angelman syndrome (AS) [MIM:105830]: A neurodevelopmental disorder characterized by severe motor and intellectual retardation, ataxia, frequent jerky limb movements and flapping of the arms and hands, hypotonia, seizures, absence of speech, frequent smiling and episodes of paroxysmal laughter, open-mouthed expression revealing the tongue. | Ubiquitin-protein ligase E3A (EC 6.3.2.-) (E6AP ubiquitin-protein ligase) (Human papillomavirus E6-associated protein) (Oncogenic protein-associated protein E6-AP) (Renal carcinoma antigen NY-REN-54) |
| Q13049 | 6.3.2.- | Limb-girdle muscular dystrophy 2H (LGMD2H) [MIM:254110]: An autosomal recessive degenerative myopathy characterized by pelvic girdle, shoulder girdle and quadriceps muscle weakness. Clinical phenotype and severity are highly variable. Disease progression is slow and most patients remain ambulatory into the sixth decade of life. Bardet-Biedl syndrome 11 (BBS11) [MIM:209900]: A syndrome characterized by usually severe pigmentary retinopathy, early-onset obesity, polydactyly, hypogenitalism, renal malformation and mental retardation. Secondary features include diabetes mellitus, hypertension and congenital heart disease. Bardet-Biedl syndrome inheritance is autosomal recessive, but three mutated alleles (two at one locus, and a third at a second locus) may be required for clinical manifestation of some forms of the disease. | E3 ubiquitin-protein ligase TRIM32 (EC 6.3.2.-) (72 kDa Tat-interacting protein) (Tripartite motif-containing protein 32) (Zinc finger protein HT2A) |
| Q13064 | 6.3.2.- | Precocious puberty, central 2 (CPPB2) [MIM:615346]: A condition defined as the development of secondary sexual characteristics in boys and girls at a chronological age that is 2.5 standard deviations below the mean age at onset of puberty in the population. Central precocious puberty results from premature activation of the hypothalamic-pituitary-gonadal axis. | Probable E3 ubiquitin-protein ligase makorin-3 (EC 6.3.2.-) (RING finger protein 63) (Zinc finger protein 127) |
| Q13085 | 6.4.1.2; 6.3.4.14 | Acetyl-CoA carboxylase 1 deficiency (ACACAD) [MIM:613933]: An inborn error of de novo fatty acid synthesis associated with severe brain damage, persistent myopathy and poor growth. | Acetyl-CoA carboxylase 1 (ACC1) (EC 6.4.1.2) (ACC-alpha) [Includes: Biotin carboxylase (EC 6.3.4.14)] |
| Q13114 | 6.3.2.- | Herpes simplex encephalitis 3 (HSE3) [MIM:614849]: A rare complication of human herpesvirus 1 (HHV-1) infection, occurring in only a small minority of HHV-1 infected individuals. HSE is characterized by hemorrhagic necrosis of parts of the temporal and frontal lobes. Onset is over several days and involves fever, headache, seizures, stupor, and often coma, frequently with a fatal outcome. | TNF receptor-associated factor 3 (EC 6.3.2.-) (CAP-1) (CD40 receptor-associated factor 1) (CRAF1) (CD40-binding protein) (CD40BP) (LMP1-associated protein 1) (LAP1) |
| Q15031 | 6.1.1.4 | Perrault syndrome 4 (PRLTS4) [MIM:615300]: A sex-influenced disorder characterized by sensorineural deafness in both males and females, and ovarian dysgenesis in females. Affected females have primary amenorrhea, streak gonads, and infertility, whereas affected males show normal pubertal development and are fertile. | Probable leucine--tRNA ligase, mitochondrial (EC 6.1.1.4) (Leucyl-tRNA synthetase) (LeuRS) |
| Q15046 | 6.1.1.6 | Charcot-Marie-Tooth disease, recessive, intermediate type, B (CMTRIB) [MIM:613641]: A form of Charcot-Marie-Tooth disease, a disorder of the peripheral nervous system, characterized by progressive weakness and atrophy, initially of the peroneal muscles and later of the distal muscles of the arms. Recessive intermediate forms of Charcot-Marie-Tooth disease are characterized by clinical and pathologic features intermediate between demyelinating and axonal peripheral neuropathies, and motor median nerve conduction velocities ranging from 25 to 45 m/sec. Deafness, autosomal recessive, 89 (DFNB89) [MIM:613916]: A form of non-syndromic deafness characterized by bilateral, prelingual, moderate to severe hearing loss affecting all frequencies. | Lysine--tRNA ligase (EC 6.1.1.6) (Lysyl-tRNA synthetase) (LysRS) |
| Q4G176 | 6.2.1.- | Combined malonic and methylmalonic aciduria (CMAMMA) [MIM:614265]: A metabolic disease characterized by malonic and methylmalonic aciduria, with urinary excretion of much larger amounts of methylmalonic acid than malonic acid, in the presence of normal malonyl-CoA decarboxylase activity. Clinical features include coma, ketoacidosis, hypoglycemia, failure to thrive, microcephaly, dystonia, axial hypotonia and/or developmental delay, and neurologic manifestations including seizures, psychiatric disease and/or cognitive decline. | Acyl-CoA synthetase family member 3, mitochondrial (EC 6.2.1.-) |
| Q5JPH6 | 6.1.1.17 | Combined oxidative phosphorylation deficiency 12 (COXPD12) [MIM:614924]: An autosomal recessive, mitochondrial, neurologic disorder characterized by onset in infancy of hypotonia and delayed psychomotor development, or early developmental regression, associated with T2-weighted hyperintensities in the deep cerebral white matter, brainstem, and cerebellar white matter. Serum lactate is increased due to a defect in mitochondrial respiration. There are 2 main phenotypic groups: those with a milder disease course and some recovery of skills after age 2 years, and those with a severe disease course resulting in marked disability. | Probable glutamate--tRNA ligase, mitochondrial (EC 6.1.1.17) (Glutamyl-tRNA synthetase) (GluRS) |
| Q5JTZ9 | 6.1.1.7 | Combined oxidative phosphorylation deficiency 8 (COXPD8) [MIM:614096]: A mitochondrial disease characterized by a lethal infantile hypertrophic cardiomyopathy, generalized muscle dysfunction and some neurologic involvement. The liver is not affected. | Alanine--tRNA ligase, mitochondrial (EC 6.1.1.7) (Alanyl-tRNA synthetase) (AlaRS) |
| Q5T160 | 6.1.1.19 | Pontocerebellar hypoplasia 6 (PCH6) [MIM:611523]: A disorder characterized by an abnormally small cerebellum and brainstem, infantile encephalopathy, generalized hypotonia, lethargy and poor feeding. Recurrent apnea, intractable seizures occur early in the course of this condition. | Probable arginine--tRNA ligase, mitochondrial (EC 6.1.1.19) (Arginyl-tRNA synthetase) (ArgRS) |
| Q63HN8 | 6.3.2.- | Moyamoya disease 2 (MYMY2) [MIM:607151]: A progressive cerebral angiopathy characterized by bilateral intracranial carotid artery stenosis and telangiectatic vessels in the region of the basal ganglia. The abnormal vessels resemble a 'puff of smoke' (moyamoya) on cerebral angiogram. Affected individuals can develop transient ischemic attacks and/or cerebral infarction, and rupture of the collateral vessels can cause intracranial hemorrhage. Hemiplegia of sudden onset and epileptic seizures constitute the prevailing presentation in childhood, while subarachnoid bleeding occurs more frequently in adults. | E3 ubiquitin-protein ligase RNF213 (EC 6.3.2.-) (ALK lymphoma oligomerization partner on chromosome 17) (Mysterin) (RING finger protein 213) |
| Q6P1M0 | 6.2.1.- | Ichthyosis prematurity syndrome (IPS) [MIM:608649]: A keratinization disorder characterized by complications in the second trimester of pregnancy resulting from polyhydramnion, with premature birth of a child with thick caseous desquamating epidermis, respiratory complications and transient eosinophilia. After recovery during the first months of life, the symptoms are relatively benign and the patients suffer from a lifelong non-scaly ichthyosis with atopic manifestations. | Long-chain fatty acid transport protein 4 (FATP-4) (Fatty acid transport protein 4) (EC 6.2.1.-) (Solute carrier family 27 member 4) |
| Q6PI48 | 6.1.1.12 | Leukoencephalopathy with brainstem and spinal cord involvement and lactate elevation (LBSL) [MIM:611105]: Autosomal recessive disease and is defined on the basis of a highly characteristic constellation of abnormalities observed by magnetic resonance imaging and spectroscopy. Affected individuals develop slowly progressive cerebellar ataxia, spasticity, and dorsal column dysfunction, sometimes with a mild cognitive deficit or decline. | Aspartate--tRNA ligase, mitochondrial (EC 6.1.1.12) (Aspartyl-tRNA synthetase) (AspRS) |
| Q6UWE0 | 6.3.2.- | Charcot-Marie-Tooth disease 2P (CMT2P) [MIM:614436]: An axonal form of Charcot-Marie-Tooth disease, a disorder of the peripheral nervous system, characterized by progressive weakness and atrophy, initially of the peroneal muscles and later of the distal muscles of the arms. Charcot-Marie-Tooth disease is classified in two main groups on the basis of electrophysiologic properties and histopathology: primary peripheral demyelinating neuropathies (designated CMT1 when they are dominantly inherited) and primary peripheral axonal neuropathies (CMT2). Neuropathies of the CMT2 group are characterized by signs of axonal degeneration in the absence of obvious myelin alterations, normal or slightly reduced nerve conduction velocities, and progressive distal muscle weakness and atrophy. Nerve conduction velocities are normal or slightly reduced. | E3 ubiquitin-protein ligase LRSAM1 (EC 6.3.2.-) (Leucine-rich repeat and sterile alpha motif-containing protein 1) (Tsg101-associated ligase) (hTAL) |
| Q6VVB1 | 6.3.2.- | Epilepsy, progressive myoclonic 2 (EPM2) [MIM:254780]: An autosomal recessive and severe form of adolescent-onset progressive epilepsy. Typically, as seizures increase in frequency, cognitive function declines towards dementia, and affected individuals die usually within 10 years after onset. EPM2 occurs worldwide, but it is particularly common in the mediterranean countries of southern Europe and northern Africa, in southern India and in the Middle East. At the cellular level, it is characterized by accumulation of starch-like polyglucosans called Lafora bodies (LBs) that are most abundant in organs with the highest glucose metabolism: brain, heart, liver and skeletal muscle. Among other conditions involving polyglucosans, EPM2 is unique in that the inclusions are in neuronal dendrites but not axons and the forming polyglucosan fibrils are associated with the endoplasmic reticulum. | E3 ubiquitin-protein ligase NHLRC1 (EC 6.3.2.-) (Malin) (NHL repeat-containing protein 1) |
| Q7Z3V4 | 6.3.2.- | Blepharophimosis-ptosis-intellectual disability syndrome (BPIDS) [MIM:615057]: A disorder characterized by blepharophimosis, ptosis, mild upslanting of the palpebral fissures, epicanthus, ectodermal anomalies, developmental delay, and severe intellectual disability with absent speech. Proportionate growth retardation with a small head circumference/microcephaly, congenital malformations, muscular hypotonia, anomalies on brain imaging with hypoplasia of the corpus callosum, and low cholesterol levels are variably present. | Ubiquitin-protein ligase E3B (EC 6.3.2.-) |
| Q7Z6Z7 | 6.3.2.- | Mental retardation, X-linked, syndromic, Turner type (MRXST) [MIM:300706]: A syndrome characterized by the association of mental retardation with macrocephaly and variable contractures. Mental retardation, X-linked 17 (MRX17) [MIM:300705]: A disorder characterized by significantly below average general intellectual functioning associated with impairments in adaptive behavior and manifested during the developmental period. Intellectual deficiency is the only primary symptom of non-syndromic X-linked mental retardation, while syndromic mental retardation presents with associated physical, neurological and/or psychiatric manifestations. | E3 ubiquitin-protein ligase HUWE1 (EC 6.3.2.-) (ARF-binding protein 1) (ARF-BP1) (HECT, UBA and WWE domain-containing protein 1) (Homologous to E6AP carboxyl terminus homologous protein 9) (HectH9) (Large structure of UREB1) (LASU1) (Mcl-1 ubiquitin ligase E3) (Mule) (Upstream regulatory element-binding protein 1) (URE-B1) (URE-binding protein 1) |
| Q86YT6 | 6.3.2.- | Left ventricular non-compaction 7 (LVNC7) [MIM:615092]: A disease due to an arrest of myocardial morphogenesis. It is characterized by a hypertrophic left ventricle with deep trabeculations and with poor systolic function, with or without associated left ventricular dilation. In some cases, it is associated with other congenital heart anomalies. | E3 ubiquitin-protein ligase MIB1 (EC 6.3.2.-) (DAPK-interacting protein 1) (DIP-1) (Mind bomb homolog 1) (Zinc finger ZZ type with ankyrin repeat domain protein 2) |
| Q8IU81 | 6.3.2.- |  | Interferon regulatory factor 2-binding protein 1 (IRF-2-binding protein 1) (IRF-2BP1) (Probable E3 ubiquitin-protein ligase IRF2BP1) (EC 6.3.2.-) |
| Q8IUD6 | 6.3.2.- | Macrocephaly, macrosomia, facial dysmorphism syndrome (MMFD) [MIM:614192]: An autosomal dominant disorder characterized by the association of macrothrombocytopathy and progressive sensorineural hearing loss without renal dysfunction. | E3 ubiquitin-protein ligase RNF135 (EC 6.3.2.-) (RIG-I E3 ubiquitin ligase) (REUL) (RING finger protein 135) (Riplet) |
| Q8IVU3 | 6.3.2.- |  | Probable E3 ubiquitin-protein ligase HERC6 (EC 6.3.2.-) (HECT domain and RCC1-like domain-containing protein 6) |
| Q8IWV7 | 6.3.2.- | Johanson-Blizzard syndrome (JBS) [MIM:243800]: This disorder includes congenital exocrine pancreatic insufficiency, multiple malformations such as nasal wing aplasia, and frequent mental retardation. Pancreas of individuals with JBS do not express UBR1 and show intrauterine-onset destructive pancreatitis. | E3 ubiquitin-protein ligase UBR1 (EC 6.3.2.-) (N-recognin-1) (Ubiquitin-protein ligase E3-alpha-1) (Ubiquitin-protein ligase E3-alpha-I) |
| Q8IYW5 | 6.3.2.- | Riddle syndrome (RIDDLES) [MIM:611943]: Characterized by increased radiosensitivity, immunodeficiency, mild motor control and learning difficulties, facial dysmorphism, and short stature. Defects are probably due to impaired localization of TP53BP1 and BRCA1 at DNA lesions. | E3 ubiquitin-protein ligase RNF168 (hRNF168) (EC 6.3.2.-) (RING finger protein 168) |
| Q8WU17 | 6.3.2.- | Renal cell carcinoma (RCC) [MIM:144700]: Renal cell carcinoma is a heterogeneous group of sporadic or hereditary carcinoma derived from cells of the proximal renal tubular epithelium. It is subclassified into clear cell renal carcinoma (non-papillary carcinoma), papillary renal cell carcinoma, chromophobe renal cell carcinoma, collecting duct carcinoma with medullary carcinoma of the kidney, and unclassified renal cell carcinoma. Clear cell renal cell carcinoma is the most common subtype. | E3 ubiquitin-protein ligase RNF139 (EC 6.3.2.-) (RING finger protein 139) (Translocation in renal carcinoma on chromosome 8 protein) |
| Q96J02 | 6.3.2.- | Syndromic multisystem autoimmune disease (SMAD) [MIM:613385]: A disorder characterized by organomegaly, failure to thrive, developmental delay, dysmorphic features and autoimmune inflammatory cell infiltration of the lungs, liver and gut. | E3 ubiquitin-protein ligase Itchy homolog (Itch) (EC 6.3.2.-) (Atrophin-1-interacting protein 4) (AIP4) (NFE2-associated polypeptide 1) (NAPP1) |
| Q96JP5 | 6.3.2.- |  | E3 ubiquitin-protein ligase ZFP91 (EC 6.3.2.-) (Zinc finger protein 757) (Zinc finger protein 91 homolog) (Zfp-91) |
| Q96K19 | 6.3.2.- | Ataxia, sensory, 1, autosomal dominant (SNAX1) [MIM:608984]: A rare disease characterized by progressive ataxia caused by degeneration of the posterior columns of the spinal cord. Affected individuals have a reduced ability to feel pain, temperature and vibration, particularly in the hands and feet. Their most prominent feature is an ataxic gait resulting from a severe loss of proprioception. Thus, patients rely on visual cues for maintaining proper body posture, such that they are unable to remain upright if their eyes are closed (Romberg sign). | E3 ubiquitin-protein ligase RNF170 (EC 6.3.2.-) (Putative LAG1-interacting protein) (RING finger protein 170) |
| Q96LR5 | 6.3.2.19 |  | Ubiquitin-conjugating enzyme E2 E2 (EC 6.3.2.19) (UbcH8) (Ubiquitin carrier protein E2) (Ubiquitin-protein ligase E2) |
| Q96MF7 | 6.3.2.- |  | E3 SUMO-protein ligase NSE2 (EC 6.3.2.-) (MMS21 homolog) (hMMS21) (Non-structural maintenance of chromosomes element 2 homolog) (Non-SMC element 2 homolog) |
| Q96PM5 | 6.3.2.- |  | RING finger and CHY zinc finger domain-containing protein 1 (EC 6.3.2.-) (Androgen receptor N-terminal-interacting protein) (CH-rich-interacting match with PLAG1) (E3 ubiquitin-protein ligase Pirh2) (RING finger protein 199) (Zinc finger protein 363) (p53-induced RING-H2 protein) (hPirh2) |
| Q96PU4 | 6.3.2.- |  | E3 ubiquitin-protein ligase UHRF2 (EC 6.3.2.-) (Np95/ICBP90-like RING finger protein) (Np95-like RING finger protein) (Nuclear protein 97) (Nuclear zinc finger protein Np97) (RING finger protein 107) (Ubiquitin-like PHD and RING finger domain-containing protein 2) (Ubiquitin-like-containing PHD and RING finger domains protein 2) |
| Q96PU5 | 6.3.2.- |  | E3 ubiquitin-protein ligase NEDD4-like (EC 6.3.2.-) (NEDD4.2) (Nedd4-2) |
| Q96RQ3 | 6.4.1.4 | Methylcrotonoyl-CoA carboxylase 1 deficiency (MCC1D) [MIM:210200]: An autosomal recessive disorder of leucine catabolism. The phenotype is variable, ranging from neonatal onset with severe neurological involvement to asymptomatic adults. There is a characteristic organic aciduria with massive excretion of 3-hydroxyisovaleric acid and 3-methylcrotonylglycine, usually in combination with a severe secondary carnitine deficiency. | Methylcrotonoyl-CoA carboxylase subunit alpha, mitochondrial (MCCase subunit alpha) (EC 6.4.1.4) (3-methylcrotonyl-CoA carboxylase 1) (3-methylcrotonyl-CoA carboxylase biotin-containing subunit) (3-methylcrotonyl-CoA:carbon dioxide ligase subunit alpha) |
| Q9HCC0 | 6.4.1.4 | Methylcrotonoyl-CoA carboxylase 2 deficiency (MCC2D) [MIM:210210]: An autosomal recessive disorder of leucine catabolism. The phenotype is variable, ranging from neonatal onset with severe neurological involvement to asymptomatic adults. There is a characteristic organic aciduria with massive excretion of 3-hydroxyisovaleric acid and 3-methylcrotonylglycine, usually in combination with a severe secondary carnitine deficiency. | Methylcrotonoyl-CoA carboxylase beta chain, mitochondrial (MCCase subunit beta) (EC 6.4.1.4) (3-methylcrotonyl-CoA carboxylase 2) (3-methylcrotonyl-CoA carboxylase non-biotin-containing subunit) (3-methylcrotonyl-CoA:carbon dioxide ligase subunit beta) |
| Q9HCE7 | 6.3.2.- |  | E3 ubiquitin-protein ligase SMURF1 (hSMURF1) (EC 6.3.2.-) (SMAD ubiquitination regulatory factor 1) (SMAD-specific E3 ubiquitin-protein ligase 1) |
| Q9HCI7 | 6.3.2.- |  | E3 ubiquitin-protein ligase MSL2 (EC 6.3.2.-) (Male-specific lethal 2-like 1) (MSL2-like 1) (Male-specific lethal-2 homolog) (MSL-2) (Male-specific lethal-2 homolog 1) (RING finger protein 184) |
| Q9HCM9 | 6.3.2.- |  | E3 ubiquitin-protein ligase TRIM39 (EC 6.3.2.-) (RING finger protein 23) (Testis-abundant finger protein) (Tripartite motif-containing protein 39) |
| Q9NP81 | 6.1.1.11 | Hyperuricemia pulmonary hypertension renal failure and alkalosis (HUPRA) [MIM:613845]: A multisystem disorder characterized by onset in infancy of progressive renal failure leading to electrolyte imbalances, metabolic alkalosis, pulmonary hypertension, hypotonia, and delayed development. Affected individuals are born prematurely. | Serine--tRNA ligase, mitochondrial (EC 6.1.1.11) (SerRSmt) (Seryl-tRNA synthetase) (SerRS) (Seryl-tRNA(Ser/Sec) synthetase) |
| Q9NPC3 | 6.3.2.- |  | E3 ubiquitin-protein ligase CCNB1IP1 (EC 6.3.2.-) (Cyclin-B1-interacting protein 1) (Human enhancer of invasion 10) |
| Q9NPD8 | 6.3.2.19 |  | Ubiquitin-conjugating enzyme E2 T (EC 6.3.2.19) (Cell proliferation-inducing gene 50 protein) (Ubiquitin carrier protein T) (Ubiquitin-protein ligase T) |
| Q9NQ86 | 6.3.2.- |  | E3 ubiquitin-protein ligase TRIM36 (EC 6.3.2.-) (RING finger protein 98) (Tripartite motif-containing protein 36) (Zinc-binding protein Rbcc728) |
| Q9NR09 | 6.3.2.- |  | Baculoviral IAP repeat-containing protein 6 (EC 6.3.2.-) (BIR repeat-containing ubiquitin-conjugating enzyme) (BRUCE) (Ubiquitin-conjugating BIR domain enzyme apollon) (APOLLON) |
| Q9NR19 | 6.2.1.1 |  | Acetyl-coenzyme A synthetase, cytoplasmic (EC 6.2.1.1) (Acetate--CoA ligase) (Acetyl-CoA synthetase) (ACS) (AceCS) (Acyl-CoA synthetase short-chain family member 2) (Acyl-activating enzyme) |
| Q9NRF8 | 6.3.4.2 |  | CTP synthase 2 (EC 6.3.4.2) (CTP synthetase 2) (UTP--ammonia ligase 2) |
| Q9NS56 | 6.3.2.- | Retinitis pigmentosa 31 (RP31) [MIM:609923]: A retinal dystrophy belonging to the group of pigmentary retinopathies. Retinitis pigmentosa is characterized by retinal pigment deposits visible on fundus examination and primary loss of rod photoreceptor cells followed by secondary loss of cone photoreceptors. Patients typically have night vision blindness and loss of midperipheral visual field. As their condition progresses, they lose their far peripheral visual field and eventually central vision as well. | E3 ubiquitin-protein ligase Topors (EC 6.3.2.-) (SUMO1-protein E3 ligase Topors) (Topoisomerase I-binding RING finger protein) (Topoisomerase I-binding arginine/serine-rich protein) (Tumor suppressor p53-binding protein 3) (p53-binding protein 3) (p53BP3) |
| Q9NW38 | 6.3.2.- | Fanconi anemia complementation group L (FANCL) [MIM:614083]: A disorder affecting all bone marrow elements and resulting in anemia, leukopenia and thrombopenia. It is associated with cardiac, renal and limb malformations, dermal pigmentary changes, and a predisposition to the development of malignancies. At the cellular level it is associated with hypersensitivity to DNA-damaging agents, chromosomal instability (increased chromosome breakage) and defective DNA repair. | E3 ubiquitin-protein ligase FANCL (EC 6.3.2.-) (Fanconi anemia group L protein) (Fanconi anemia-associated polypeptide of 43 kDa) (FAAP43) |
| Q9NWF9 | 6.3.2.- | Cerebellar ataxia and hypogonadotropic hypogonadism (CAHH) [MIM:212840]: A disease characterized by cerebellar symptoms and signs of sex steroid deficiency. Clinical features include cerebellar and brain stem atrophy, cerebellar ataxia, hypothalamic LHRH deficiency, hypogonadotrophic hypogonadism, lack of secondary sexual characteristics, and infertility. | E3 ubiquitin-protein ligase RNF216 (EC 6.3.2.-) (RING finger protein 216) (Triad domain-containing protein 3) (Ubiquitin-conjugating enzyme 7-interacting protein 1) (Zinc finger protein inhibiting NF-kappa-B) |
| Q9P2R7 | 6.2.1.5 | Mitochondrial DNA depletion syndrome 5 (MTDPS5) [MIM:612073]: A disorder due to mitochondrial dysfunction. It is characterized by infantile onset of hypotonia, neurologic deterioration, a hyperkinetic-dystonic movement disorder, external ophthalmoplegia, deafness, variable renal tubular dysfunction, and mild methylmalonic aciduria in some patients. | Succinyl-CoA ligase [ADP-forming] subunit beta, mitochondrial (EC 6.2.1.5) (ATP-specific succinyl-CoA synthetase subunit beta) (Renal carcinoma antigen NY-REN-39) (Succinyl-CoA synthetase beta-A chain) (SCS-betaA) |
| Q9UPN9 | 6.3.2.- | Thyroid papillary carcinoma (TPC) [MIM:188550]: A common tumor of the thyroid that typically arises as an irregular, solid or cystic mass from otherwise normal thyroid tissue. Papillary carcinomas are malignant neoplasm characterized by the formation of numerous, irregular, finger-like projections of fibrous stroma that is covered with a surface layer of neoplastic epithelial cells. | E3 ubiquitin-protein ligase TRIM33 (EC 6.3.2.-) (Ectodermin homolog) (RET-fused gene 7 protein) (Protein Rfg7) (Transcription intermediary factor 1-gamma) (TIF1-gamma) (Tripartite motif-containing protein 33) |
| Q9Y252 | 6.3.2.- | Esophageal cancer (ESCR) [MIM:133239]: A malignancy of the esophagus. The most common types are esophageal squamous cell carcinoma and adenocarcinoma. Cancer of the esophagus remains a devastating disease because it is usually not detected until it has progressed to an advanced incurable stage. | E3 ubiquitin-protein ligase RNF6 (EC 6.3.2.-) |
| Q9Y2Z4 | 6.1.1.1 | Myopathy with lactic acidosis and sideroblastic anemia 2 (MLASA2) [MIM:613561]: A rare oxidative phosphorylation disorder specific to skeletal muscle and bone marrow. Affected individuals manifest sideroblastic anemia, progressive lethargy, muscle weakness, and exercise intolerance associated with persistent lactic acidemia. | Tyrosine--tRNA ligase, mitochondrial (EC 6.1.1.1) (Tyrosyl-tRNA synthetase) (TyrRS) |

***Table S1: List of the ligase proteins in humans that have polymorphisms associated with human diseases.****Taken from the homo_sapiens_variation.txt file from UniProtKB (14^th^ Feb 2014).*

| **EC Class and (chemistry type)** | **List of EC numbers** |
| --- | --- |
| EC 6.1.c.d (C-O bond formation) | 6.1.1.1, 6.1.1.2, 6.1.1.3, 6.1.1.4, 6.1.1.5, 6.1.1.6, 6.1.1.7, 6.1.1.9, 6.1.1.10, 6.1.1.11, 6.1.1.12, 6.1.1.13, 6.1.1.14, 6.1.1.15, 6.1.1.16, 6.1.1.17, 6.1.1.18, 6.1.1.19, 6.1.1.20, 6.1.1.21, 6.1.1.22, 6.1.1.23, 6.1.1.24, 6.1.1.25, 6.1.1.26 |
| EC 6.2.c.d (C-S bond formation) | 6.2.1.1, 6.2.1.4, 6.2.1.5, 6.2.1.6, 6.2.1.7, 6.2.1.8, 6.2.1.9, 6.2.1.11, 6.2.1.12, 6.1.2.13, 6.1.2.14, 6.2.1.15, 6.2.1.16, 6.2.1.17, 6.2.1.18, 6.2.1.22, 6.2.1.23, 6.2.1.24, 3.2.1.25, 6.2.1.26, 6.2.1.27, 6.2.1.28, 6.2.1.30, 6.2.1.61, 6.2.1.33 |
| EC 6.3.c.d (C-N bond formation) | 6.3.1.1, 6.3.1.2, 6.3.1.4, 6.3.1.5, 6.3.1.6, 6.3.1.7, 6.3.1.8, 6.3.1.9, 6.3.1.10, 6.3.1.11  6.3.2.1, 6.3.2.2, 6.3.2.3, 6.3.2.4, 6.3.2.5, 6.3.2.6, 6.3.2.7, 6.3.2.8, 6.3.2.9, 6.3.2.10, 6.3.2.11, 6.3.2.13, 6.3.2.16, 6.3.2.20, 6.3.2.23, 6.3.2.24, 6.3.2.25, **6.3.2.26, 6.3.2.27**  6.3.3.1, 6.3.3.2, 6.3.3.3, 6.3.3.4  6.3.4.1, 6.3.4.2, 6.3.4.3, 6.3.4.4, 6.3.4.5, 6.3.4.6, 6.3.4.7**, 6.3.4.8**, 6.3.4.9, 6.3.4.10, 6.3.4.11, 6.3.4.12, 6.3.4.13, 6.3.4.14, 6.3.4.15, **6.3.4.16**, 6.3.4.17, 6.3.4.18  **6.3.5.1**, **6.3.5.2**, **6.3.5.3**, **6.3.5.6**, **6.3.5.7**, **6.3.5.9** |
| EC 6.4.c.d (C-C bond formation) | 6.4.1.1, 6.4.1.2, 6.4.1.3, 6.4.1.4, 6.4.1.5, 6.4.1.6, 6.4.1.7 |
| EC 6.5.c.d (P-O bond formation) | 6.5.1.4 |
| EC 6.6.c.d (Metal ligation) | 6.6.1.1 |

***Table S2: list of EC numbers in Fig.2 in the order that they appear.***

*EC numbers highlighted in Fig. 2 are highlighted in the same colour in the table: blue for ligases that utilise multiple ATP molecules and green for the Gln-dependent ligases.*

*
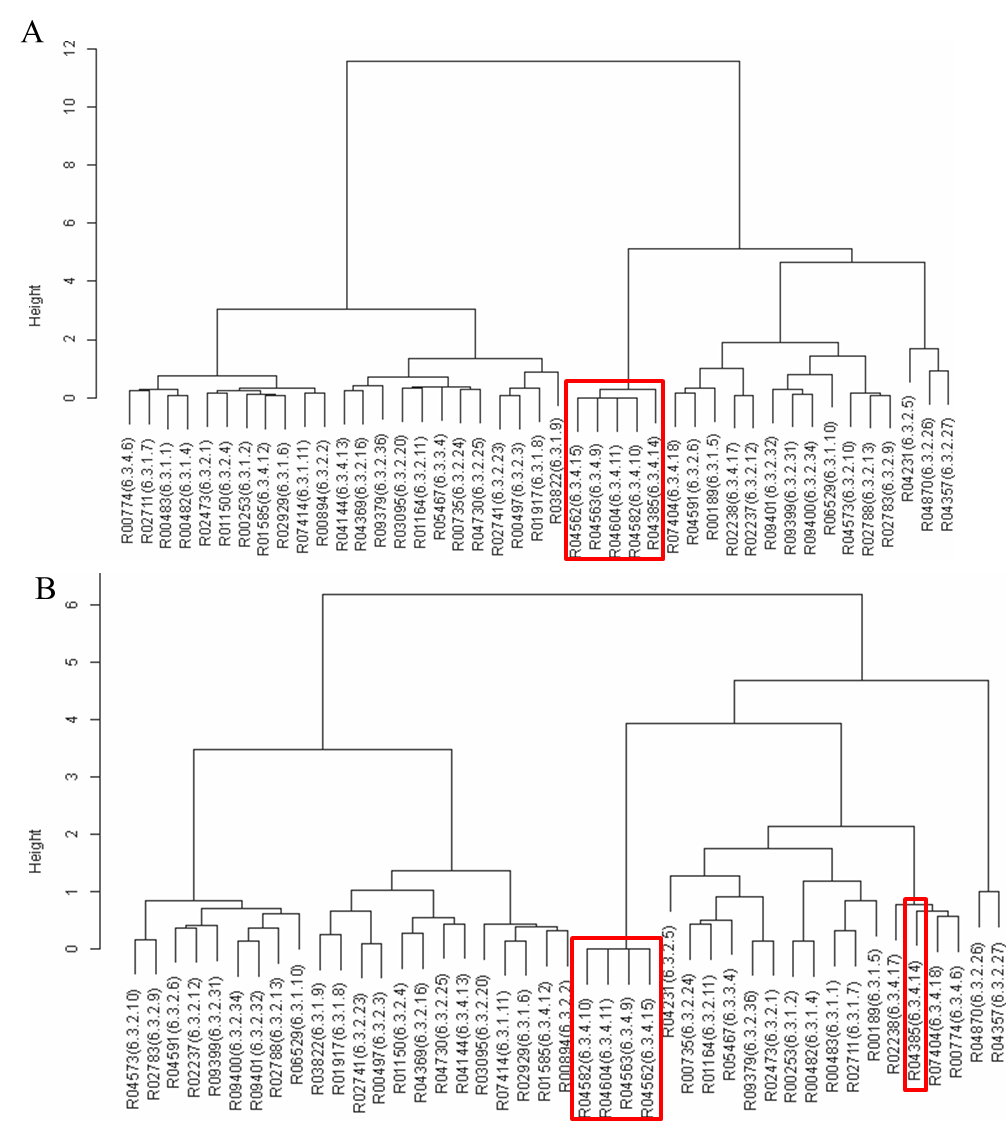
*

*Figure S2: Hierarchical clustering trees of the 6.3.c.d “simple” C-N bond forming ligase reactions. A) The substrate similarity tree; the red box represents the five ligases involving a biotin molecule as a substrate. B) The Reaction centre similarity tree; the red boxes represent the two different reaction centre types for these five enzymes with biotin as a substrate. The first set (6.3.2.10, 11, 9 and 15) add biotin to another enzyme, EC 6.3.4.14 adds a carboxyl group to the biotin molecule at the opposite end to the first set. The R numbers represent the KEGG reaction identifier used to identify the EC reaction, the EC number is shown in parentheses after this identifier.*

| **EC Number** | **Reaction Figure** |
| --- | --- |
| 6.1.1.13 | 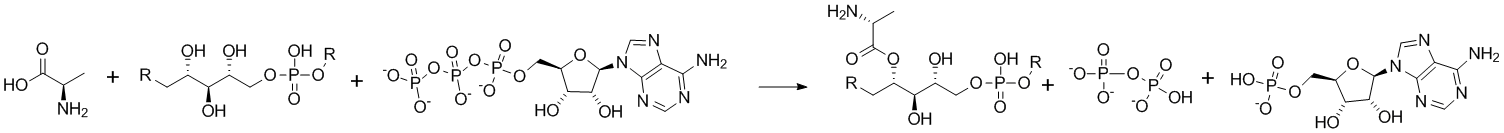 |
| 6.2.1.1 | 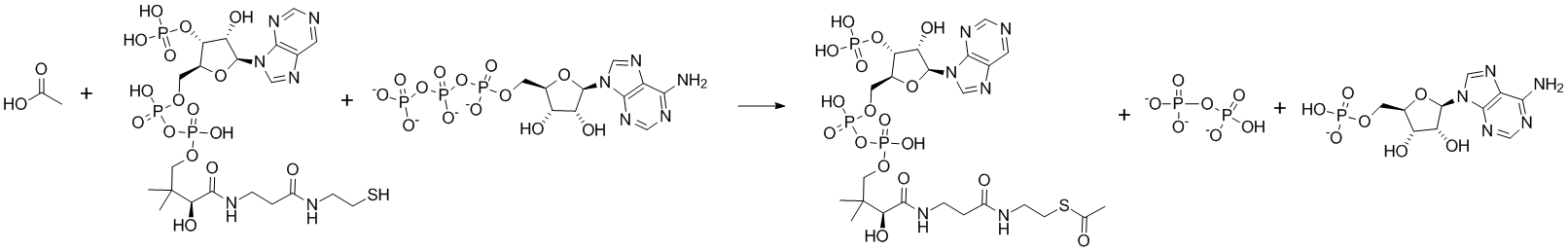 |
| 6.2.1.2 | 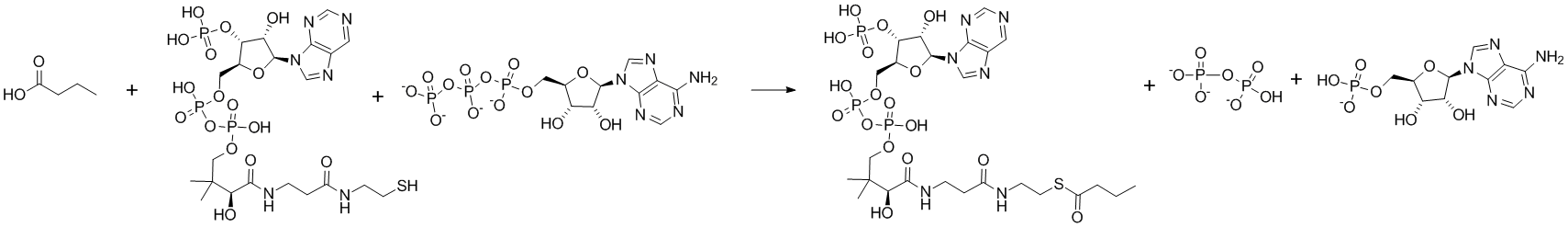 |
| 6.2.1.3 | 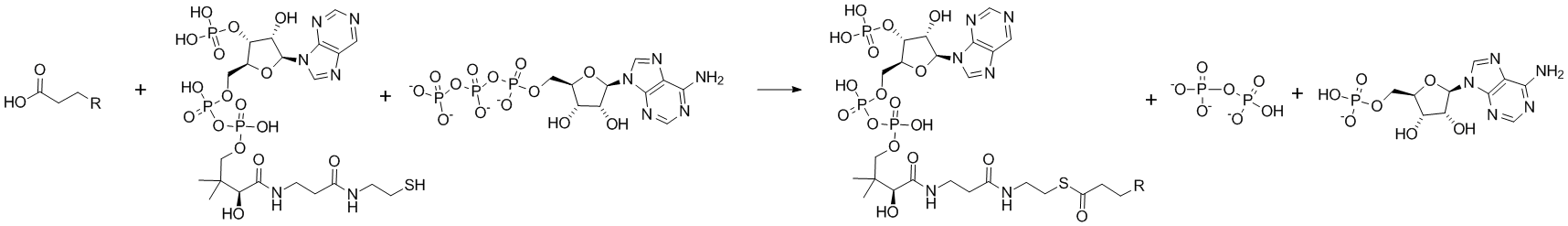 |
| 6.2.1.7 | 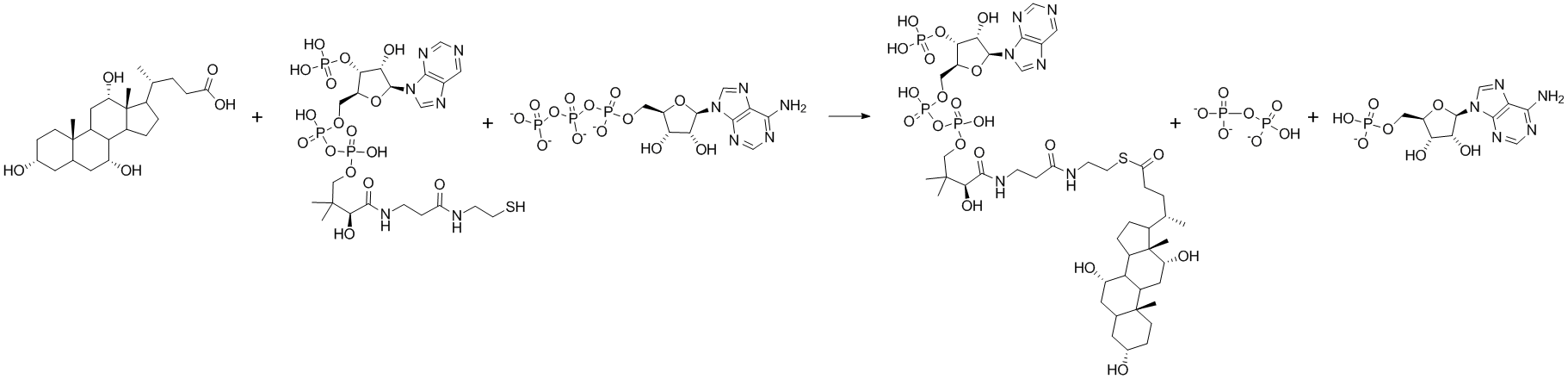 |
| 6.2.1.12 | 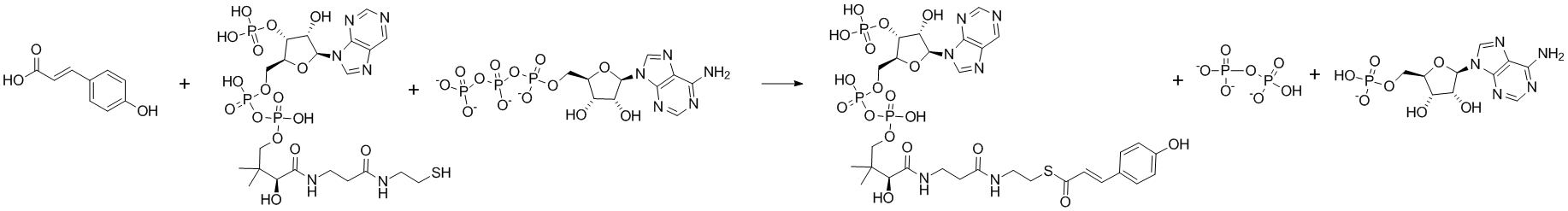 |
| 6.2.1.16 | 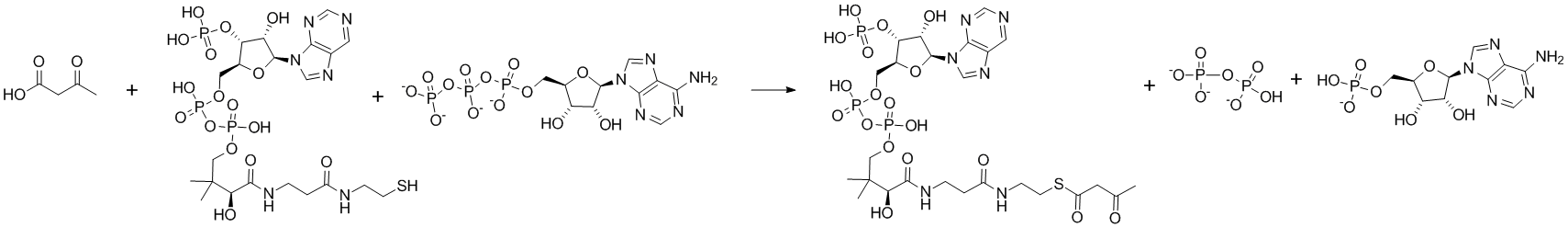 |
| 6.2.1.17 | 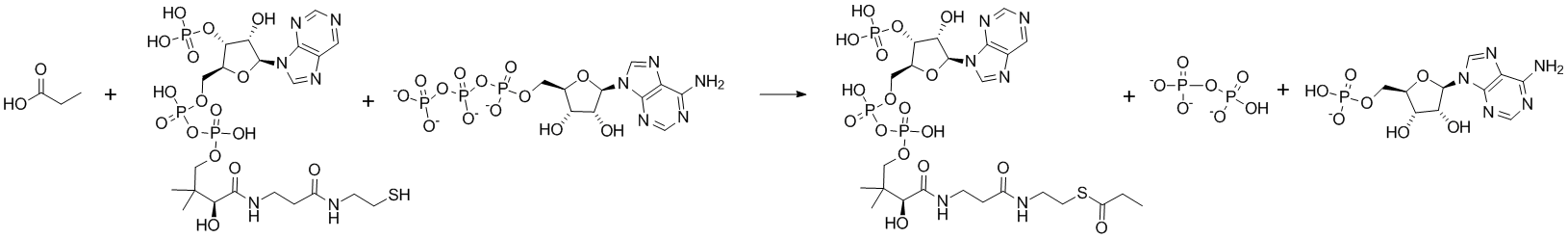 |
| 6.2.1.20 | 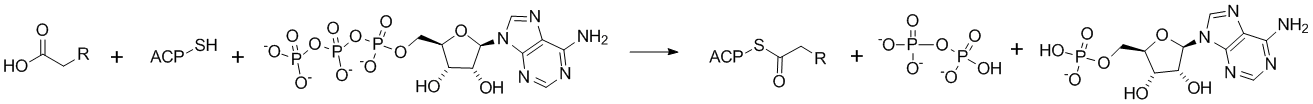 |
| 6.2.1.25 | 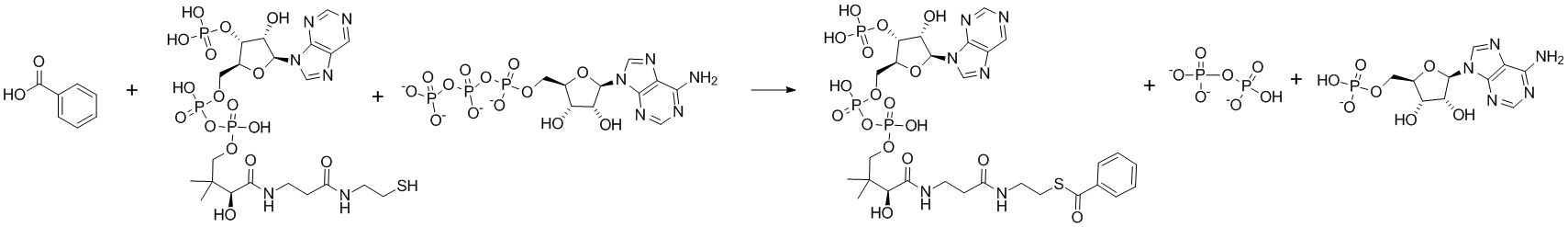 |
| 6.2.1.26 | 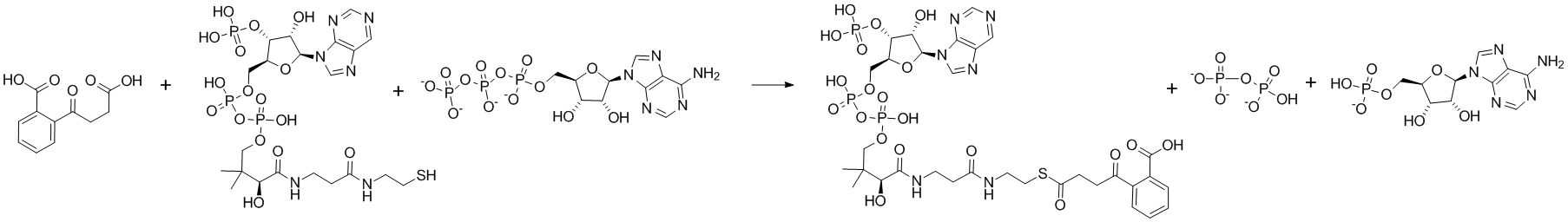 |
| 6.2.1.27 | 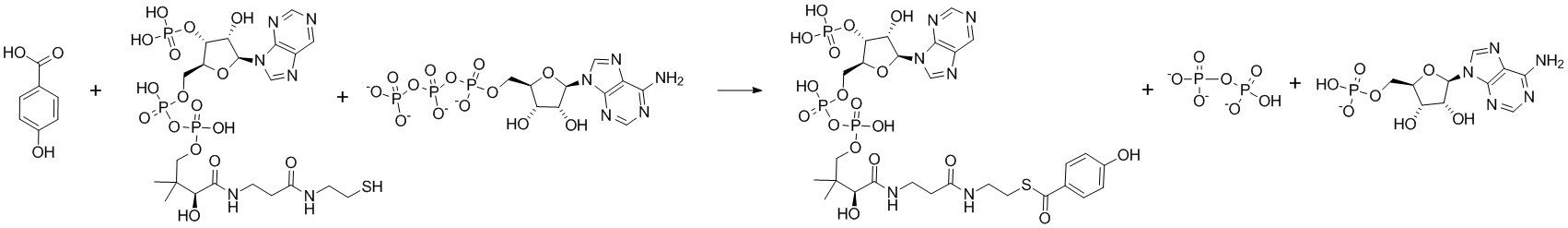 |
| 6.2.1.33 | 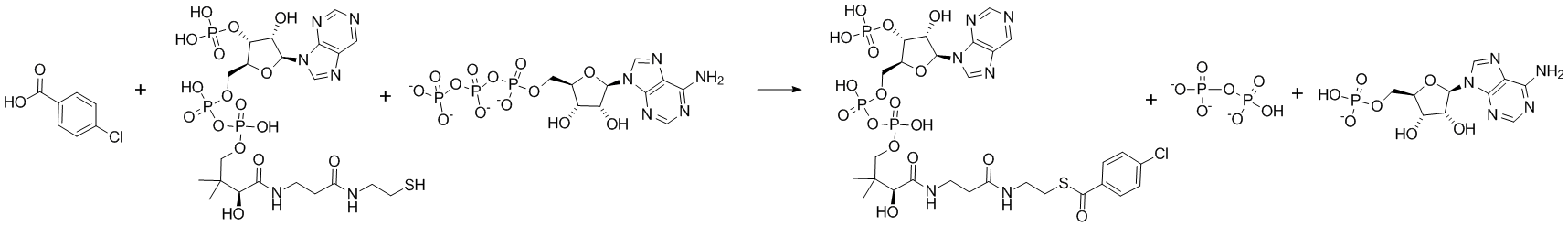 |
| 6.2.1.36 | 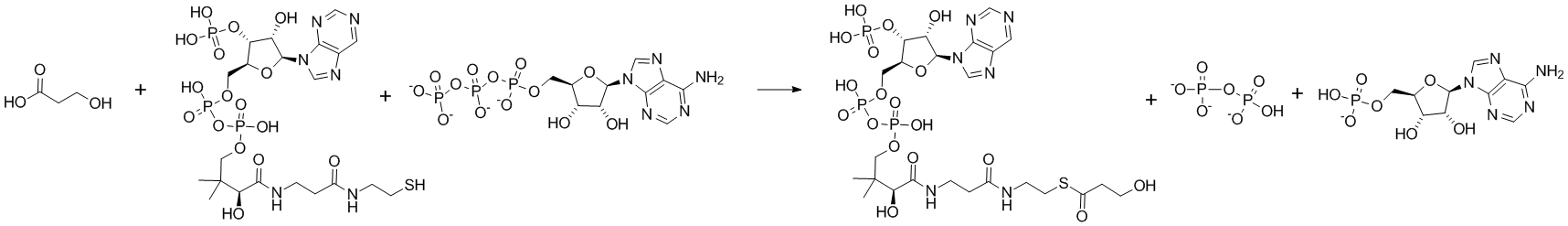 |
| 6.2.1.37 | 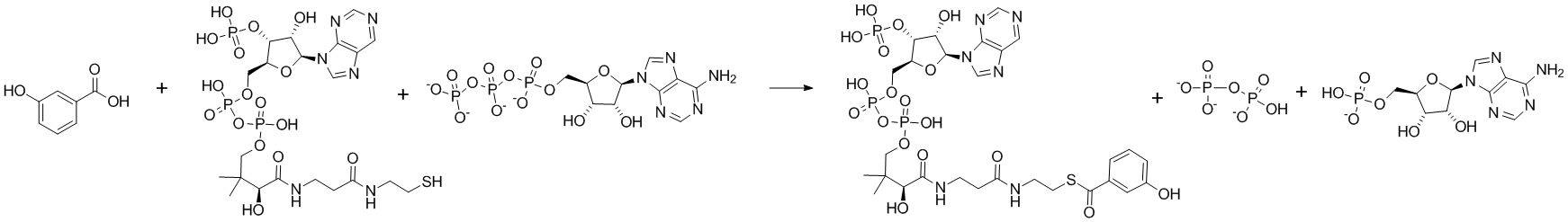 |
| 6.3.2.26 | 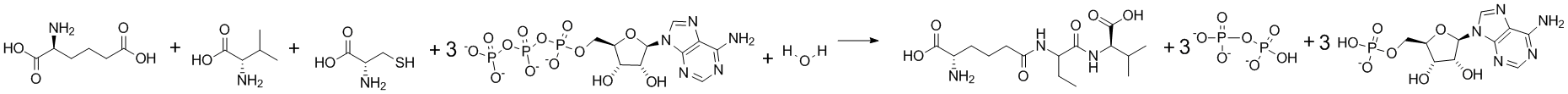 |

***Table S3: The EC numbers and corresponding overall reactions in the Acyl-CoA ligase cluster.***


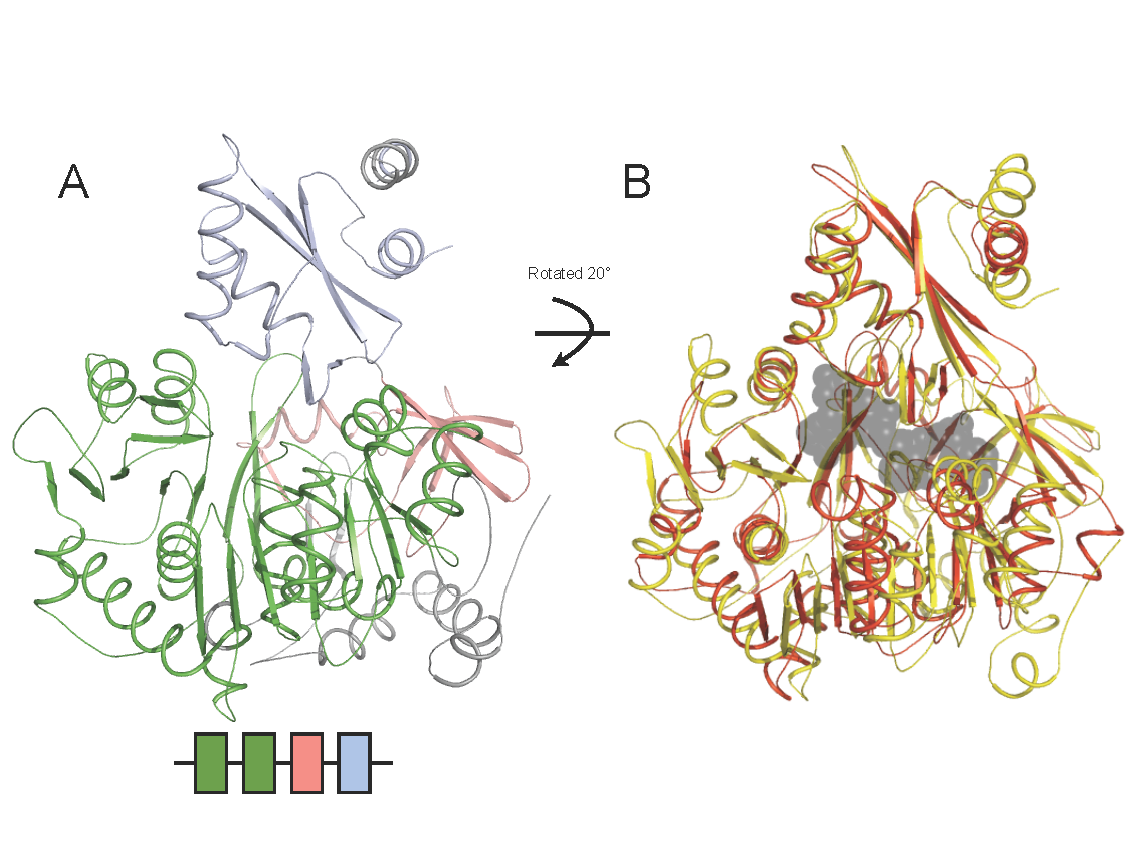


*Figure S3: Example of a structural motif that has evolved to perform different chemical functions: C-S and C-O bond formation.* ***A.*** *The multi domain architecture mapped on to a structure of Acetyl-CoA synthetase (EC 6.2.1.1) using the same colouring as in the MDA graph of Figure 7: green – CATH ID 3.40.50.980; red – CATH ID 2.30.38.10; blue – CATH ID 3.30.300.30.* ***B.*** *Structures of Acetyl-CoA synthetase (EC 6.2.1.1; PDB ID 1PG4) in yellow and D-alanine:D-alanyl carrier protein ligase (EC 6.1.1.13; PDB ID 3E7W) in red superimposed to each other with the CoA and AMP ligands that are co-crystallized with the structure of Acetyl-CoA synthetase shown as space filled spheres.*


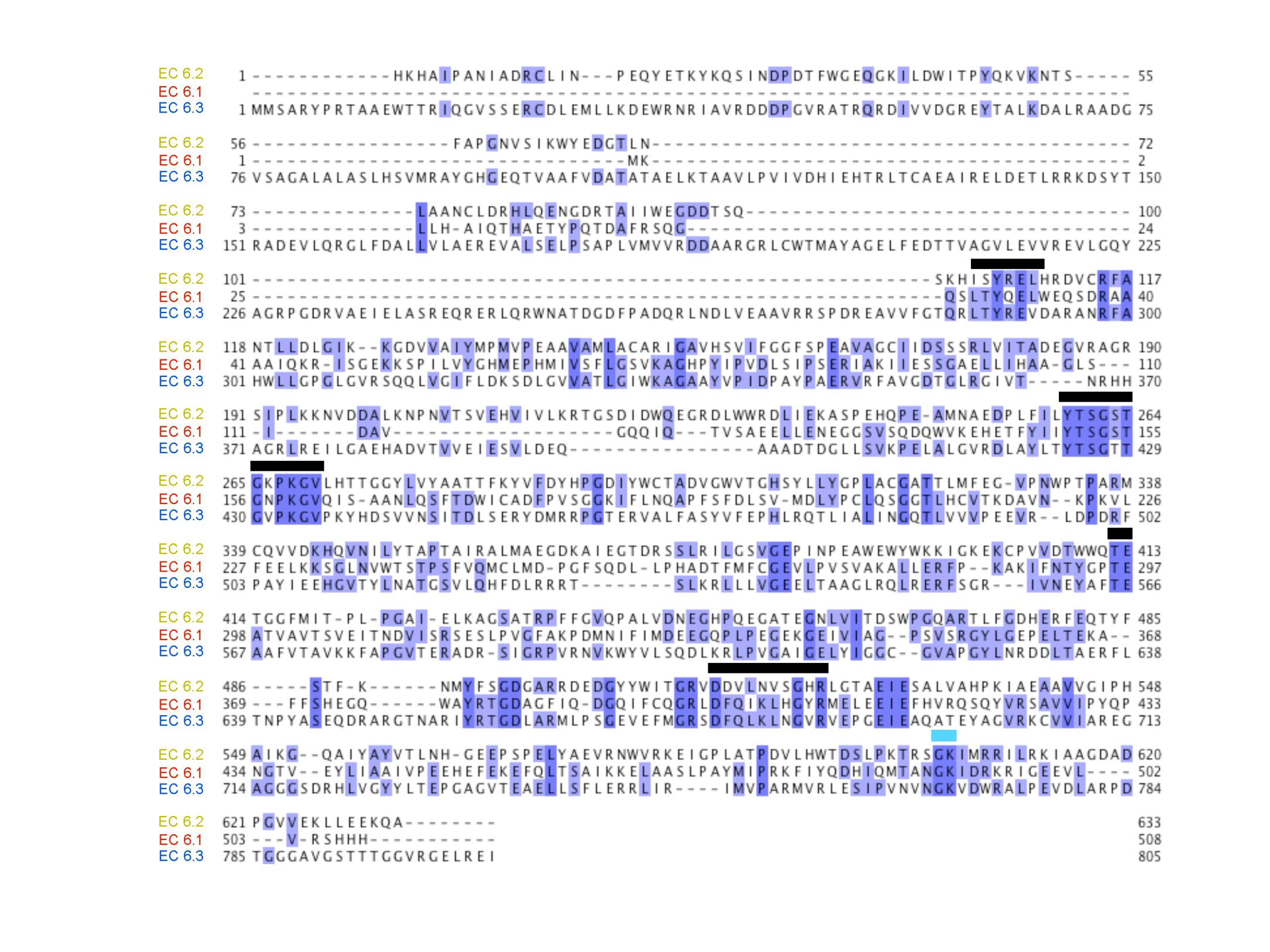


*Figure S4: The structurally informed multiple sequence alignment of the two structures (PDB ID 1PG4 and 3E7W) used for modelling sequence of N-(5-amino-5-carboxypentanoyl)-L-cysteinyl-D-valine synthase (UniProtKB ID Q01757). Conserved motifs involved in ligand binding and the active site are highlighted by a black bar above the relevant sequence motif. Sequence similarity is shown by the intensity of the blue colouring. All sequences have less than 20% sequence identity to each other.*

***D-alanine—poly(phosphoribitol) ligase (EC 6.1.1.13)***

The overall reaction relating to this EC number is actually the product of four gene products (DltA to DltD), which are responsible for the incorporation of D-Ala into lipoteichoic acid in the cell walls of gram positive bacteria[^1^](#_ENREF_1) (Supplementary Fig S5). The sequences we have clustered relate to the DltA gene product, which is thought to be responsible for the initial step in the alanylation of lipoteichioc acid in bacterial cell walls. In this step the D-Ala substrate is ligated onto the phosphopanthinyl group of D-Ala carrier protein (DltC). This complex is then transported to the cell wall at which point the D-Ala is transferred to the lipoteichoic acid. This final step of the reaction is not well understood and it remains unclear if DltA remains associated with the complex and performs the final step of the reaction as well.

*Figure S5: The role of the four gene products DltA to DltD in the incorporation of D-alanine into lipoteichoic acid in the cell walls of gram positive bacteria. The NTP portion of the reaction is shown in cyan. The proposed reaction scheme for as D-alanine—poly(phosphoribitol) ligase (EC 6.1.1.13) is shown in the dotted box*

***N-(5-amino-5-carboxypentanoyl)-L-cysteinyl-D-valine synthase (EC 6.3.2.26)***

The overall reaction for this enzyme is significantly different to the other reactions in the set (see Fig. 7B). However, closer inspection of the postulated mechanism (Fig. S6) shows that the three amino acid substrates are initially ligated to AMP, then Cys and Val are joined and after an isomerisation where L-Val is changed to D-Val, the dipeptide is added to a 4’-phosphopantetheine cofactor. This complex is then added to the final amino acid before being hydrolysed off[^2^](#_ENREF_2).

*Figure S6: The proposed reaction scheme for N-(5-amino-5-carboxypentanoyl)-L-cysteinyl-D-valine synthase (EC 6.3.2.26). The NTP portion of the reaction is coloured in cyan, the conserved cofactor moiety is shown in blue and the bonds formed are shown in red*.

*Figure S7: The conserved reactive motif (shown in blue) in all the reactions of the Acyl—CoA ligase cluster from Fig. 7.*

**References**

1. Yonus, H., Neumann, P., Zimmermann, S., May, J. J., Marahiel, M. A. & Stubbs, M. T. (2008). Crystal structure of DltA. Implications for the reaction mechanism of non-ribosomal peptide synthetase adenylation domains. J Biol Chem 283, 32484-91.

2. Marahiel, M. A., Stachelhaus, T. & Mootz, H. D. (1997). Modular Peptide Synthetases Involved in Nonribosomal Peptide Synthesis. Chem Rev 97, 2651-2674.
